# Volatile-mediated interactions between phylogenetically different soil bacteria

**DOI:** 10.3389/fmicb.2014.00289

**Published:** 2014-06-11

**Authors:** Paolina Garbeva, Cornelis Hordijk, Saskia Gerards, Wietse de Boer

**Affiliations:** ^1^Department Microbial Ecology, Netherlands Institute of Ecology (NIOO-KNAW)Wageningen, Netherlands; ^2^Department of Soil Quality, Wageningen University and Research CentreWageningen, Netherlands

**Keywords:** bacterial volatiles, inter-specific interactions, transcriptional responses, sand microcosm, infochemicals

## Abstract

There is increasing evidence that organic volatiles play an important role in interactions between micro-organisms in the porous soil matrix. Here we report that volatile compounds emitted by different soil bacteria can affect the growth, antibiotic production and gene expression of the soil bacterium *Pseudomonas fluorescens* Pf0–1. We applied a novel cultivation approach that mimics the natural nutritional heterogeneity in soil in which *P. fluorescens* grown on nutrient-limited agar was exposed to volatiles produced by 4 phylogenetically different bacterial isolates (*Collimonas pratensis*, *Serratia plymuthica*, *Paenibacillus* sp., and *Pedobacter* sp.) growing in sand containing artificial root exudates. Contrary to our expectation, the produced volatiles stimulated rather than inhibited the growth of *P. fluorescens*. A genome-wide, microarray-based analysis revealed that volatiles of all four bacterial strains affected gene expression of *P. fluorescens*, but with a different pattern of gene expression for each strain. Based on the annotation of the differently expressed genes, bacterial volatiles appear to induce a chemotactic motility response in *P. fluorescens*, but also an oxidative stress response. A more detailed study revealed that volatiles produced by *C. pratensis* triggered, antimicrobial secondary metabolite production in *P. fluorescens*. Our results indicate that bacterial volatiles can have an important role in communication, trophic - and antagonistic interactions within the soil bacterial community.

## Introduction

Most soil bacteria occur in multi-species communities, in which a variety of interactions influences their behavior and performance. Recent years have shown an explosion of research on “communication” between different soil bacterial species (Keller and Surette, 2006; Ryan and Dow, 2008; Shank and Kolter, 2009; Garbeva et al., 2011b). Most attention has been paid to the perception of other bacterial species via signaling compounds diffusing in liquid or semi-solid media. However, an important characteristic of most soils is the occurrence of air-filled pores. Hence, the gaseous phase forms an integral part of the natural surroundings of soil microorganisms. It has been estimated that the area of soil particles covered by microorganisms is less than 1% implying that the distance between microbial neighbors can be considerable (Young et al., 2008). Volatile molecules can act over a wider range of scale than non-volatiles as they can diffuse through both the liquid and gaseous phases of the soil (Effmert et al., 2012). Therefore, volatiles are thought to play an important role in communication and competition between physically separated soil microorganisms (Kai et al., 2009; Chernin et al., 2011; Garbeva et al., 2011a, 2014; Effmert et al., 2012).

It is well known that many soil microorganisms produce volatile organic compounds (VOC). In a recent review by Effmert et al. (2012) an overview is given of the wide variety of volatiles emitted by bacterial strains isolated from soils. From this review as well as from other papers it is clear that the spectrum of volatile compounds differs between bacterial species, even between closely related ones (Groenhagen et al., 2013; Garbeva et al., 2014). In addition, environmental conditions, in particular nutrient availability, do influence the composition of bacterial volatiles (Blom et al., 2011; Garbeva et al., 2014).

With respect to the functioning of soil microbial volatiles, most attention has been given to suppressive effects of bacterial volatiles on soil eukaryotes that are harmful to agricultural crops, e.g., plant-pathogenic fungi and plant-parasitic nematodes (Gu et al., 2007; Kai et al., 2007; Zou et al., 2007; Verginer et al., 2010; Garbeva et al., 2014). However, the role of volatiles in interactions between soil bacterial species has been hardly studied. Given the physically separated distribution of bacterial populations (micro-colonies) in the porous soil matrix we hypothesize that volatiles play key roles in interspecific bacterial interactions. In the current study, our aim was to test volatile-mediated interactions between soil bacterial species under conditions that are realistic to soil conditions. To this end we applied a novel cultivation approach where we tried to mimic volatile-mediated interactions between bacteria in the rhizosphere and bacteria outside the rhizopshere. As model bacteria we selected five phylogenetically different soil isolates that do occur in natural rhizosphere communities. The main research questions we addressed were (1) Do rhizobacteria protect their “territory” against potential rhizosphere invaders by producing volatiles that suppress bacteria outside the rhizosphere or (2) Can bacteria outside the rhizosphere profit from the volatiles produced by rhizosphere-inhabiting bacteria? Our expectation was that rhizosphere-inhabiting bacteria will invest part of the energy obtained from metabolizing root-exudates in the production of suppressing volatiles.

## Materials and methods

### Bacterial isolates and growth media used in this work

*Collimonas pratensis* Ter91 (β-Proteobacteria), *Paenibacillus* sp. P4 (Bacilli) and *Pedobacter* sp. V48 (Sphingobacteria) have been isolated isolates from the rhizopshere of Marram grass in sandy dune soils in The Netherlands (De Boer et al., 1998, 2004); *Serratia plymuthica* PRI-2C strain (Y-Proteobacteria) was isolated from maize rhizosphere, The Netherlands (Garbeva et al., 2004) *Pseudomonas fluorescens* Pf0–1 was isolated from an agricultural soil in Massachusetts, USA (Compeau et al., 1988). All strains were pre-cultured from frozen glycerol stocks on 1/10 strength Tryptone Soya Broth agar (CMO129, Oxoid).

### Bioassay for testing the effect of bacterial volatiles on pseudomonas fluorescens Pf0–1

The bioassay was performed as described in the Figure S1. The top area of the glass Petri dish contained 12 ml water-agar medium (20 g L^−1^ of Agar, 5 g L^−1^ of NaCl, 1 g L^−1^ of KH_2_PO_4_ and 0.1 g L^−1^ (NH_4_)_2_SO_4_; pH 6.5_._). This carbon-limited medium was used to represent the situation in the bulk soil where bacterial growth is limited by availability of easily degradable carbon compounds.The water-agar medium was inoculated with *Pseudomonas fluorescens* Pf0–1 of which 5.0 × 10^6^ cells were spread over the water-agar surface. The bottom area of the glass Petri dish contained 45 g of sterile washed sea sand (Honeywell Specialty Chemicals Seelze GmbH, Germany) supplemented with 4.5 ml artificial root exudates and bacterial inoculum (3.0 × 10^6^/gr sand) from monocultures of *Collimonas pratensis* Ter 91; *Paenibacillus* sp. P4; *Pedobacter* sp. V48; *Serratia plymuthica* PRI-2C or a mixture of these soil bacteria. As control treatment *P. fluorescens* Pf0–1 was exposed only to sand with artificial root exudates without bacterial inoculum. The artificial root exudates (ARE) stock solution contained 18.4 mM glucose; 18.4 mM fructose; 9.2 mM saccharose; 4.6 mM citric acid; 9.2 mM lactic acid; 6.9 mM succinic acid; 18.4 mM L-serine; 11 mM L-glutamic acid and 18.4 mM L-alanine (C/N 10.4). To each plate 4.5 ml of ARE working solution consisting of 1.5 ml of stock solution mixed with 3 ml of 10 mM phosphate buffer (pH 6.5) was added as described in Baudoin et al. (2003). The plates were incubated at 20°C while packed in aluminium foil. After 3 days of incubation bacterial numbers in the top and bottom compartments were determined. *P. fluorescens* Pf0–1 cells that had developed on the top water-agar area were scraped and suspended in 3 ml 10 mM phosphate buffer (pH 6.5). One hundred and fifty micro liter of this bacterial suspension was used for OD measurements and plating of serial dilutions on 1/10 TSBA medium (the remaining 2.85 ml were used for RNA extraction, see below). For enumeration of bacteria growing in the bottom area 1 g of sand was taken from each plate and transferred into a 20 ml Greiner tube. Ten milli liter of 10 mM phosphate buffer (pH 6.5) were added and the tubes were shaken on a rotary shaker at 350 rpm for 30 min at 20°C. Subsequently, serial dilutions were plated in triplicate on 1/10 TSBA. All plates were incubated at 20°C and bacterial colonies were counted after 48 h.

### Transcriptional analysis

For total RNA extraction all suspensions retrieved from agar (see above) were diluted in sterile phosphate buffer to the same optical density (OD; 600 nm) to obtain equal amounts of cells for RNA extraction. The cell suspensions were centrifuged at 16,000 × g for 3 min. RNA was extracted from the cell pellets with the Artrum Total RNA Mini Kit (BIO-RAD cat# 732-6820) according to the manufacturer's recommendations. The extracted total RNA was treated with the TURBO DNA-free Kit to remove DNA (Ambion cat#1907).

Transcriptomic analyses were performed using high-density, multiplex (12x72K) microarrays designed and produced by Roche NimbleGen (Cat# A7241-00-01). Arrays consisted of 60-mer probes covering 5735 genes, 6 probes per gene, 2 replicates. cDNA synthesis, labeling of cDNA with Cy3 dye and hybridization were performed by the Micro Array Department (MAD), University of Amsterdam, The Netherlands (www.microarray.nl).

Each treatment and control were performed in triplicates. The lists of differential expressed genes were extracted by comparison of each interaction with the control. The Robust Microarray Analysis (RMA)-normalized gene expression data were analyzed with the Array Star 2 software for microarray analysis (DNASTAR, Madison, Wisconsin, USA). Analysis was performed, with application of false discovery rate (FDR; *Benjamini Hochberg*) and multiple testing corrections.

Quantitative RT-PCR was performed to verify the gene expression detected by microarray analysis. First strand cDNA was synthesized with random hexamer primers from Invitrogen (cat# 48190-011) using SuperScript™ Double-Stranded cDNA Synthesis kits (Invitrogen cat#11917-010). Two μL of cDNA was subjected to real-time PCR using SYBR Green PCR master mix (Applied Biosystem). For each target gene [five differentially expressed genes: catalase; sulfotransferase, methyl-accepting chemotaxis sensory transducer, cytochrome C oxidase, chemotaxis sensory transducer and two non-differentially expressed control housekeeping genes: 16S rRNA and RNA polymerase (rpoB)], forward and reverse primers were designed using Primer Express software (PE Applied Biosystem, Warrington, UK). All primers used for real-time PCR were first tested using conventional PCR with DNA isolated from *P. fluorescens* Pf0–1. Real-time PCR was performed using a Corbett Research Rotor-Gene 3000 thermal cycler (Westburg, Leusden, The Netherlands) with the following conditions: initial cycle 95°C for 15 min and 40 cycles of: 95°C for 15 s; 56°C for 50 s and 72°C for 50 s. The relative expression of the genes was normalized to that of the house keeping genes.

### Bacterial volatiles trapping and GC/MS analysis

For the collection of bacterial volatiles that were produced in sand containing artificial root exudates, glass Petri dishes with leads, to which a steel trap containing 150 mg Tenax TA and 150 mg Carbopack B (Markes International Ltd., Llantrisant, UK) could be fixed, were used (Figure S1b). Volatiles were collected during 72 h of incubation at 20°C, traps were removed, capped and stored at 4°C until analysis.

Volatiles were desorbed from the traps using an automated thermodesorption unit (model Unity, Markes International Ltd., Llantrisant, UK) at 200°C for 12 min (He flow 30 ml/min). The trapped volatiles were introduced into the GC-MS (model Trace, ThermoFinnigan, Austin, TX, USA) by heating the cold trap for 3 min to 270°C. Split ratio was set to 1:4, and the column used was a 30 × 0.32 mm ID RTX-5 Silms, film thickness 0.33 μm (Restek, Bellefonte, PA, USA). Temperature program used was as follows: from 40 to 95°C at 3°C/min, then to 165°C at 2°C/min, and finally to 250°C at 15°C/min. The VOCs were detected by the MS operating at 70 eV in EI mode. Mass spectra were acquired in full scan mode (33–300 AMU, 0.4 scan/s). Compounds were identified by their mass spectra using deconvolution software (AMDIS) in combination with NIST 2008 (National Institute of Standards and Technology, USA, http://www.nist.gov) and Wiley 7th edition spectral libraries and by their linear retention indexes (lri).

The lri values were compared with those found in the NIST and the NIOO lri database. Mass spectra and lri values for identification were also checked by analysis of pure compounds.

### Test of pure individual volatiles

Several volatiles produced by *Collimonas pratensis* Ter91 and *Serratia plymuthica* PRI-2C were commercially available. A number of these compounds, namely methanthiosulfonate (CH_3_SO_2_SCH_3_); S-methyl thioacetate (C_3_H_6_OS); dimethyldisulfide (CH_3_S_2_CH_3_) and benzonitrile (C_6_H_5_CN), were tested for their individual effect on *P. fluorescens* Pf0–1 growth. Each volatile was applied in concentrations ranging from 3, 12, 30 to 60 μmol as a droplet on a filter paper on the bottom of the Petri dish. The effect of these pure compounds on *P. fluorescens* Pf0–1 growth was determined by CFU enumeration as described above.

### Extraction of secondary metabolites from P. fluorescens Pf0–1

For extraction of secondary metabolites the water-agar inoculated with *P. fluorescens* Pf0–1 was removed carefully from the plate and cut in small (1-cm-diameter) pieces. These pieces were vigorously shaken in 20 mL of 80% (v/v) acetone for 1 h at room temperature. The acetone solution was centrifuged for 10 min at 4000 × *g* and the acetone was evaporated under air flow. The water fraction was acidified with trifluoroacetic acid [0.1% (v/v)], mixed with 2 volumes of ethylacetate and shaken vigorously for 5 min. After incubation overnight at −20°C the unfrozen (ethylacetate) fraction that contains the active compounds was carefully transferred to a new flask and dried under air flow. The dried extract was dissolved in 150 μl of 50% (v/v) methanol and subjected to reverse phase high pressure liquid chromatography (RP-HPLC) analysis and test for antimicrobial activity.

The antimicrobial compounds dissolved in 50% methanol were tested for activity against the fungi *Rhizoctonia solani* AG2.2IIIB and *Fusarium oxysporum*, and the bacteria *Bacillus* sp. V102 and *Collimonas pratensis* Ter91 (as described in Garbeva et al., 2011a,b).

### Statistical analysis

All experiments were performed in triplicate with three independent replicates for each treatment and controls. ArrayStar 2 (DNASTAR, Madison, WI) was used for statistical analysis of differentially expressed genes applying Student's *t*-test with Benjamini-Hochberg false discovery rate correction. The statistical analyses of fungal biomass, bacterial enumeration, antagonistic tests and qRT-PCR were carried out with XLStat 2010 (Addinsoft, New York, USA) using a Student's *t*-Test. Data were considered to be statistically different at *p* ≤ 0.05.

## Results

### Effect of bacterial volatiles on P. fluorescens Pf0–1 growth

**Figure 1:**
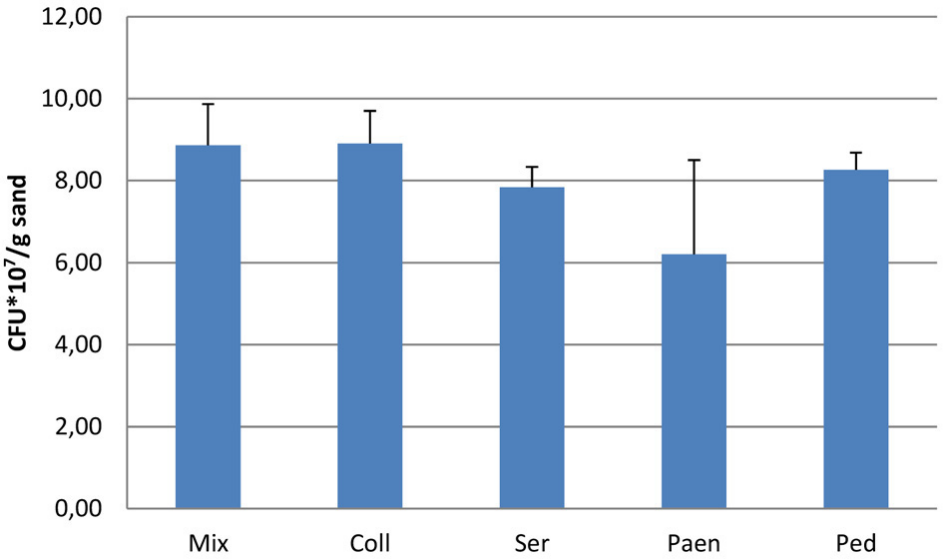
**Number of bacteria (CFUs) after 3 days of incubation in sand containing artificial root exudates**. Coll- *Collimonas pratensis* Ter 91; Ser- *Serratia plymuthica* PRI-2C; Paen- *Paenibacillus* sp. P4; Ped- *Pedobacter* sp. V48 and Mix- mix of all four bacteria. Inoculation densities were 3.0 × 10^6^/gr sand. Presented values are means of three replicates and error bars indicate standard deviation. No significant differences were found between the different bacterial inoculums.

**Figure 2:**
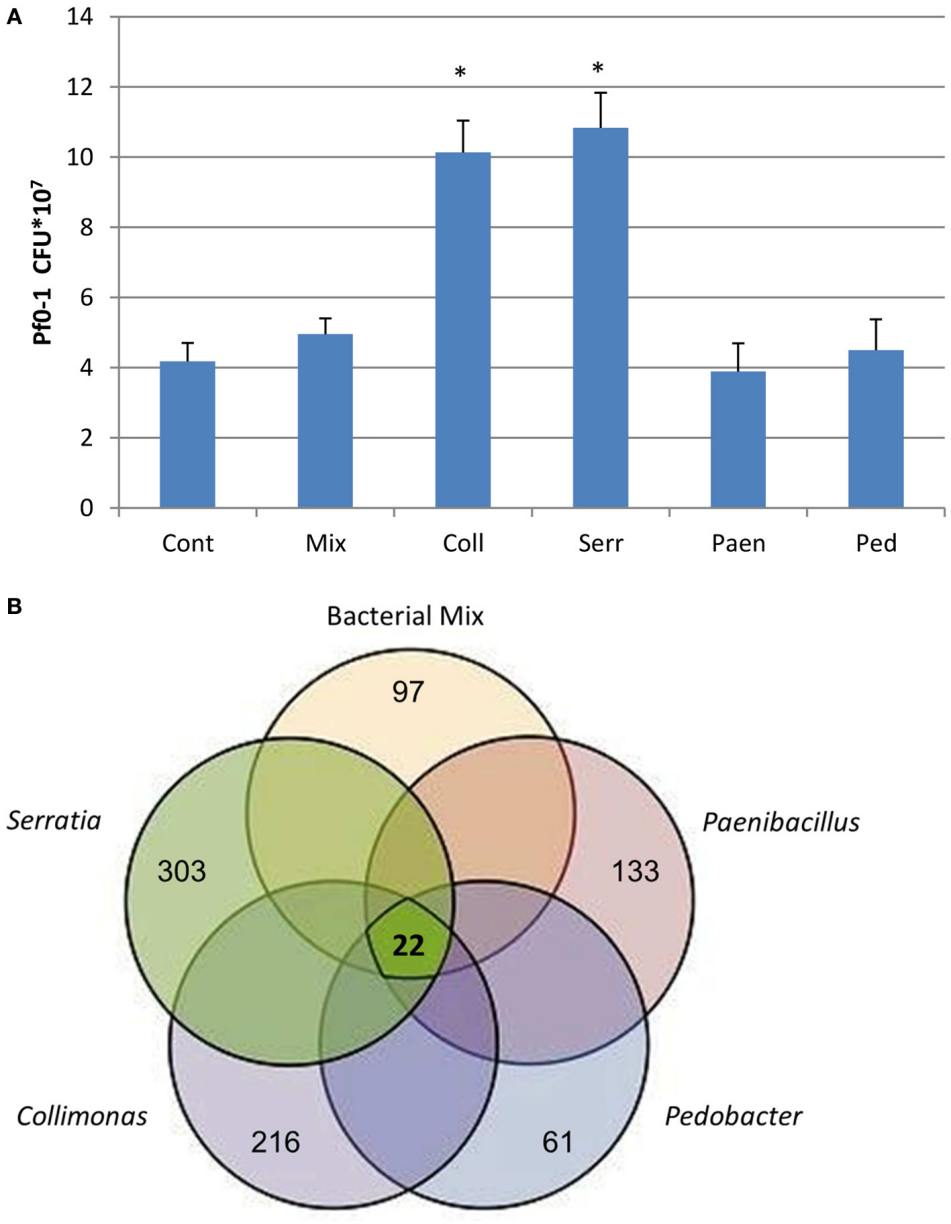
**(A)** Number of *P. fluorescens* Pf0–1 colony forming units (CFUs) developed after 3 days of incubation on water-agar while exposed to volatiles emitted by different bacteria growing in sand containing artificial root exudates: Coll- *Collimonas pratensis* Ter 91; Serr- *Serratia plymuthica* PRI-2C; Paen- *Paenibacillus* sp. P4; Ped- *Pedobacter* sp. V48 and Mix- mix of all 4 bacteria. Cont- control is sand with artificial root exudates but without bacteria. Inoculation density of *P. fluorescens* Pf0–1 is 5.0 × 10^6^ cells. Presented data are means of three replicates, error bars indicate standard deviation and ^*^indicates significant differences in CFUs between control and treatments (*P* < 0.05). **(B)** Venn diagram representing the number of differentially expressed genes in *P. fluorescens* Pf0–1 in response to volatiles emitted by different bacteria grown in sand containing artificial root exudates. The bold number in the middle of the diagram represents the common differentially expressed genes in all treatments as listed in Table 1. Other numbers indicate treatment-specific differences in gene expression: *Paenibacillus* sp. P4 total 133 genes (63 up-regulated and 70 down regulated); *Pedobacter* sp. V48 total 61 genes (50 up-regulated and 11 down regulated); *Collimonas pratensis* Ter91 total 216 genes (73 up-regulated and 143 down regulated); *Serratia plymuthica* PRI-2C total 303 genes (93 up-regulated and 210 down regulated) and bacterial mix of all 4 strains total 97 genes (31 up-regulated and 66 down regulated).

After 3 days of incubation, the four bacterial strains that were grown in sand containing artificial root exudates had reached similar cell densities (number of CFUs) (Figure 1). All four strains and also the mixture of strains produced volatiles in the sand microcosms (see next section), but the effect of these volatiles on the growth of *P. fluorescens* was different (Figure 2A). Volatiles produced by *C. pratensis* and *S. plymuthica* stimulated the growth of *P. fluorescens*, whereas volatiles emitted by *Paenibacillus* sp., *Pedobacter* sp. and the mix of all 4 bacteria did not affect *P. fluorescens* growth.

### Volatiles produced by bacteria growing in sand microcosms

GC-MS analysis revealed that besides commonly known bacterial VOCs such as dimethylsulfide, 2-pentanone, 4-heptanone, 2-heptanol, and 2-undecanone, each bacterial species produced a different blend of volatiles in sand supplied with artificial root exudates (Table 1). The highest numbers of unique volatile compounds were emitted by *C. pratensis* and *S. plymuthica*. Several of these volatiles (like S-methyl thioacetate, methyl thiocyanate, dimethyl disulfide, benzonitrile) were produced by both bacteria. *Paenibacillus* sp. and *Pedobacter* sp. produced less different volatile compounds and this was also the case for the mixture of four bacterial species. Interestingly, the volatiles produced by the bacterial mix included compounds that were not detected in the spectrum of volatiles produced by the different bacterial monocultures (like 1-tetradecanol, isopropyl dodecanoate, branched alcane, and unknowns).

**Table 1:** **Volatile organic compounds produced by 4 bacterial strains growing in sterile sand containing artificial root exudates**.

**Compound name**	**RI**
**PRODUCED IN ALL TREATMENTS**	
Dimethyl sulfide	<600
2-pentanone	688
3-pentanone	702
2,4 pentadione	779
4-heptanone	868
2-heptanol	900
beta pinene	969
nonanal	1102
2-decanone	1193
decanal	1203
2-undecanol	1301
6-dodecanone	1371
Octylcyclohexane	1443
**PRODUCED BY SERRATIA PLYMUTHICA PRI-2C**	
S-methyl thioacetate	703
Methyl thiocyanate	713
Dimethyl disulfide	740
1H-pyrrole	751
Methyl 3-methylbutanoate	775
Ethyl butanoate	802
Chlorobenzene	838
2,4 dithiapentane	882
3-heptanol	895
Dimethyl sulfone	922
Benzonitrile	978
2-octanone	989
5-dodecanone	1372
2-pentadecanone	1697
**PRODUCED BY COLLIMONAS PRATENSIS TER91**	
2- methyl propanal	<600
Ethenyl acetate	<600
S-methyl thioacetate	703
Methyl thiocyanate	713
Dimethyl disulfide	740
3-methyl 2-pentanoene	749
Methyl 2-methylbutanoate	774
Methyl 3-methylbutanoate	775
3-hexanone	789
4-methyl 3-penten-2-one	808
2-acetyl 1-pyrroline	922
Methyl hexanoate	923
3-methyl 2-heptanone	940
Benzonitrile	978
7-methyl-3-methylene-1,6-octadiene (myrcene)	987
Ethyl hexanoate	1001
Methyl 2-ethylhexanoate	1044
1-methyl 4-(1-methylethyl) 1,4-cyclohexadiene (terpinene)	1056
Methyl 2-methylbenzoate	1179
Methyl salicylate	1190
Methyl 3-methylbenzoate	1199
Methyl 4-methylbenzoate	1207
Methyl 2,6-dimethylbenzoate	1239
**PRODUCED BY *PAENIBACILLUS* SP. P4**	
3-methyl-2-hexanone	840
Pentalactone	947
Hexanoic-acid	981
Carene isomer	1007
Tridecane	1300
**PRODUCED BY *PEDOBACTER* SP. V48**	
1,3-butadiene, 2-methyl-	<600
Cyclohexanone	891
Oxime methoxy phenyl	907
Benzaldehyde	958
Camphene	940
Hexanoic-acid	981
Unknown	1145
Diphenylsulfide	1574
**PRODUCED ONLY IN BACTERIAL MIX**	
Sulfur dioxide	<600
Branched alcane	1021
Isopropyl dodecanoate	1628
Salicylic acid hexyl ester	1672
Unknown	1674
1-tetradecanol	1675

*RI- Linear retention index Rtx-5 ms column*.

*The table excludes volatiles that were also present in the controls (non-inoculated sand with artificial root exudates)*.

### Transcriptional response of P. fluorescens Pf0–1 to bacterial volatiles

Microarray-based analyses did reveal strong differences in expression of *P. fluorescens* gene when exposed to volatiles emitted by the different bacterial species (Figure 2B). Only a small set of 22 genes was differentially expressed by volatiles of all bacteria, including the mixture. These genes were mainly involved in amino acid transport and—metabolism, energy production and conversion, signal transduction mechanisms, inorganic ion transport and—metabolism, secretion and cell motility (Table 2). In addition, all exposures to bacterial volatiles resulted in increased expression of a gene encoding catalase, an enzyme involved in the protection of cells against damage by reactive oxygen species. The RT-PCR analysis of 5 selected differentially expressed genes confirmed the microarray data (Figure S2). The highest number of differentially expressed genes in *P. fluorescens* was obtained when exposed to volatiles produced by *C. pratensis*, *Paenibacillus* sp. and *S. plymuthica* (Tables S1–S3) whereas volatiles emitted by *Pedobacter* sp. and the bacterial mix affected the expression of much less genes (Tables S4, S5). There was high similarity in the effect of volatiles of *C. pratensis* and *S. plymuthica* on gene expression (116 common differentially expressed genes) of *P. fluorescens* which corresponds to the high similarity in the composition of volatiles produced by these two bacteria (Tables S1, S3).

**Table 2:** **Common genes differentially expressed in *P. fluorescens* Pf0–1 exposed to volatiles produced by four bacterial species and a mixture of these species in sand containing artificial root exudates**.

**SEQ_ID**	**Gene description**	**Fold change (1)**	**Fold change (2)**	**Fold change (3)**	**Fold change (4)**	**Fold change (5)**	**Possible**
**UP-REGULATED GENES WITH > 2-FOLD CHANGE**						
Pfl_0064	Catalase	6.3	6.7	7.1	3.6	6.4	Inorganic ion transport and metabolism
Pfl_0157	Sulfotransferase	2.6	3.2	4.3	2.3	3.1	Amino acid transport and metabolism
Pfl_0378	Methyl-accepting chemotaxis sensory transducer	3.3	3.2	3.9	2.6	2.4	Signal transduction mechanisms
Pfl_0623	Putative diguanylate cyclase (GGDEF domain)	5.4	7.3	4.1	2.7	3.1	Signal transduction mechanisms
Pfl_1076	Hypothetical protein	2.9	2.6	3.6	2.1	4.6	Function unknown
Pfl_1813	Coproporphyrinogen III oxidase	2.4	6.8	3.1	2.6	3.1	Coenzyme metabolism
Pfl_1824	Cytochrome c oxidase cbb3-type, subunit III	2.8	4.6	2.7	3.1	2.3	Energy production and conversion
Pfl_1826	Cytochrome C oxidase, mono-heme subunit/FixO	2.9	4.8	2.5	4.2	3.3	Energy production and conversion
Pfl_1827	Cytochrome c oxidase cbb3-type, subunit I	2.3	5.8	2.6	3.6	3.2	Energy production and conversion
Pfl_2904	D-isomer specific 2-hydroxyacid dehydrogenase, NAD-binding	2.1	2.7	2.7	2.4	2.4	Amino acid transport and metabolism
Pfl_2907	Chemotaxis sensory transducer	6.3	6.6	7.1	6.5	6.1	Cell motility and secretion
Pfl_4382	Chemotaxis sensory transducer	2.1	6.2	2.6	2.5	3.7	Cell motility and secretion
Pfl_4989	Aldehyde dehydrogenase (NAD+)	3.1	2.8	4.3	2.9	4.2	Energy production and conversion
Pfl_5345	Aldehyde dehydrogenase	2.7	2.1	2.6	2.4	6.5	Energy production and conversion
**DOWN-REGULATED GENES WITH > 2-FOLD CHANGE**						
Pfl_0044	Protein of unknown function DUF1328	2.1	4.1	3.2	2.2	4.2	Function unknown
Pfl_0045	Hypothetical protein	2.8	6.9	2.4	2.4	2.9	Function unknown
Pfl_1337	Amidase	2.1	5.8	5.7	1.9	4.3	Energy production and conversion
Pfl_1779	Assimilatory nitrat reductase (NADH) alpha apoprotein	4.7	3.3	2.8	2.7	5.3	Inorganic ion transport and metabolism
Pfl_1780	Assimilatory nitrite reductase NAD(P)H small subunit	5.7	2.3	3.4	3.1	5.8	Inorganic ion transport and metabolism
Pfl_1781	Nitrite and sulphite reductase 4Fe-4S region	7.1	2.5	3.6	3.2	3.3	Energy production and conversion
Pfl_4818	Transport-associated	2.8	3.6	2.7	2.3	4.5	General function prediction only
Pfl_4819	General secretion pathway protein H	1.9	2.8	3.1	2.9	3.3	Cell motility and secretion

*Fold change of differentially expressed genes in P. fluorescens Pf0–1 exposed to volatiles produced by (1) Collimonas pratensis (2) Serratia plymuthica (3) Paenibacillus sp. (4) Pedobacter sp., and (5) Mixture of four bacterial species. The differentially expressed genes were identified using the false discovery rate (Benjamini-Hochberg) correction method with 99% confidence (P < 0.05)*.

### Effect of individual volatiles on P. fluorescens Pf0–1 growth

Four volatiles produced by *C. pratensis* and *S. plymuthica* namely methanthiosulfonate; S-methyl thioacetate; dimethyldisulfide and benzonitrile, were tested individually for their effect on *P. fluorescens*. Benzonitrile and dimethyldisulfide stimulated the growth of *P. fluorescens* growth when applied in concentrations above 3μmol (Figure S3). Methanthiosulfonate and S-methyl thioacetate did not reveal any effect on the Pf0–1 growth.

### Effect of bacterial volatiles on P. fluorescens Pf0–1 secondary metabolites production

To test if bacterial volatiles triggered *P. fluorescens Pf0–1* secondary metabolites production, ethylacetate extracts were made from water-agar on which *P. fluorescens* had been grown while exposed to volatiles emitted by *C. pratensis*. Comparison of the HPLC profiles and activities of these extracts with those extracts obtained from controls revealed that *P. fluorescens* Pf0–1 produced many more secondary metabolites (11 vs. 7) with higher intensity when exposed to volatiles produced by *C. pratensis* (Figure S4). Furthermore, these extracts showed antimicrobial activity against a Gram-positive bacterium (*Bacillus* sp.) and the plant pathogenic fungus (*Fusarium oxysporum*) but did not affect growth of *C. pratensis*.

## Discussion

The role of bacterial volatiles in microbial interactions is increasingly recognized in the last years. However, most work on bacterial volatiles to date is done *in vitro* under nutrient-rich conditions (Beck et al., 2003; Chun et al., 2010; Kai et al., 2010; Weise et al., 2012; Kim et al., 2013) and may not be representative for the conditions that occur in the soil environment. In the present study, we developed an experimental set-up that is approaching a situation which is likely to occur in soils namely the volatile-mediated interactions between bacteria growing in the rhizosphere with bacteria present outside the rhizosphere. Since the latter are experiencing starvation conditions we hypothesized that volatiles produced by rhizosphere bacteria could act as a chemoattractant to the nutrient-richer conditions nearby. On the other hand production of volatiles by rhizospere-inhabiting bacteria could also be used to suppress other bacteria which would prevent invasion of the rhizosphere by potentially new competitors.

It is known that bacterial volatiles can have antimicrobial activity and inhibit the growth of other microorganisms (Kai et al., 2007, 2009; Garbeva et al., 2014). However, none of the four rhizobacteria appeared to produce volatiles that were inhibiting the starved model bacterium *P. fluorescens*. It is plausible that similar to what has been reported for effects of antibiotics, bacteria are becoming highly tolerant to volatiles when they are under nutrient limitation (Nguyen et al., 2011). Volatiles emitted by *C. pratensis* and *S. plymuthica* did even stimulate *Pseudomonas* growth and were probably used as energy source. Some volatiles produced by these two bacteria were applied as pure substances and did also result in increased *P. fluorescens* growth. Growth of microbes in the area surrounding the rhizosphere is limited by carbon availability and, therefore, carbon-containing volatiles produced by rhizosphere microbes may be important energy resources for such microbes (Owen et al., 2007). Kleinheinz et al. (1999) revealed that *P. fluorescens* were able to degrade alpha-pinene released by plants and to use it as a sole energy source.

Although bacterial volatiles did not inhibit the growth of *P. fluorescens* they caused expression of genes that indicate a stress response, e.g., Pfl_0064 Catalase. It is known that catalase can be induced under conditions of oxidative stress which may have been caused by some of the volatiles (Lushchak, 2001; Kwon et al., 2010).

The genome-wide microarray-based analyses revealed that *P. fluorescens* had a different response in gene expression to volatiles emitted by the different bacterial species. Only a small set of 22 genes was differentially expressed in all treatments. Among these common differentially expressed genes were Pfl_0064 Catalase, an important enzyme in protecting the cell against damage by reactive oxygen species; Pfl_0157 Sulfotransferase, belonging to a group of enzymes that catalyze the transfer of a sulfo group from a donor molecule to an acceptor alcohol or amine; Pfl_2907 and Pfl_4382 Chemotaxis sensory transducer genes, genes that are important for regulation of bacterial chemotaxis, and Pfl_0623 Diguanylate cyclase (GGDEF domain), a gene that has been indicated to be responsible for the wrinkly spreader phenotype in *P. fluorescens* (Malone et al., 2007; Silby et al., 2009). The difference in transcriptional response of *P. fluorescens* to different bacterial strains appeared to reflect the composition of volatiles. *C. pratensis* and *S. plymuthica*, producing similar sets of volatiles, caused similar changes in gene expression. Many differentially expressed genes were genes involved in *P. fluorescens* metabolic activity, signal transduction mechanisms, cell motility and secretion.

Soil bacteria including *Pseudomonas* possess many two-component signal transduction systems that help them to adapt to fluctuations in environmental conditions (Gao et al., 2007; Rodriguez et al., 2013; Willett et al., 2013). The set of differently expressed genes involved in two-component signal transduction was not the same for the 4 volatile-producing bacterial species (Tables S1–S5) indicating that volatiles may act as an infochemicals providing information on the identity of surrounding microorganisms. Furthermore, *C. pratensis* and *S. plymuthica* triggered expression of several genes related to chemotaxis and motility indicating that part of their volatiles may act as chemoattractants guiding *P. fluorescens* to a close-by environment with nutrient input.

Recent studies revealed that inter-specific interactions between phylogenetically unrelated soil bacteria often leads to production of antimicrobial compounds (Garbeva et al., 2011a,b; Onaka et al., 2011; Hopwood, 2013). Most antimicrobial compounds are produced in growth density-dependent manner and nutrient availability has a major impact on the expression of biosynthetic genes (Sanchez et al., 2010; Van Wezel and McDowall, 2011). Our results revealed that volatiles can have an effect on secondary metabolites production by *P. fluorescens*. When exposed to volatiles emitted by *C. pratensis, P. fluorescens* produced secondary metabolites that had inhibiting activity against a Gram positive bacterium and a fungus but not against the Gram negative volatile producer. It is plausible that the volatiles served as energy sources and/or signal inducing secondary metabolite production. The volatile-triggered antibiotic production in *P. fluorescens* could point a strategy to combine movement (chemotaxis- and motility genes) with increasing competitive strength (antibiotics) to invade into the nutrient-providing rhizosphere zone.

The volatile blend produced by soil bacteria growing in the sand microcosm containing artificial root exudates differed between different bacterial species. Several studies indicated that the numbers and spectrum of volatiles produced by bacteria depends on growth conditions and nutrient availability (Blom et al., 2011; Weise et al., 2012; Garbeva et al., 2014). Interestingly, the composition of volatiles produced by the mixture of 4 bacterial species was different from that produced by each of the bacterial monocultures which may be due to competitive interactions between the bacterial species. The blend of volatiles produced by bacterial mix had a smaller effect on the expression of genes in *P. fluorescens* than the volatiles produced by monocultures. The effect of volatiles produced by the bacterial mixture is probably more representative for the situation occurring in natural environment.

In conclusion, this work is the first to report that volatiles compounds emitted by different rhizobacteria can affect the growth and gene expression of other phylogenetically distinct and physically separated bacteria. The model bacteria *P. fluorescens* growing under nutrient limited conditions was able to sense bacterial activity based on volatile production. The results obtained here do not indicate that volatiles produced by rhizobacteria are inhibitory to the bacteria outside the rhizosphere. Bacteria outside the rhizosphere may even profit from the volatiles emitted by rhizobacteria. This work reveals novel information on the role of bacterial volatiles in long-distance microbial interactions in soil and indicates that bacterial volatiles may act as growth substrates and as infochemicals affecting gene expression, metabolism and triggering the production of other secondary metabolites in responding bacteria.

### Conflict of interest statement

The authors declare that the research was conducted in the absence of any commercial or financial relationships that could be construed as a potential conflict of interest.
