# Supplementary material for: Volatile-mediated interactions between phylogenetically different soil bacteria
Source: Front Microbiol. 2014 Jun 11;5:289. doi: 10.3389/fmicb.2014.00289 (PMC4052926; doi:10.3389/fmicb.2014.00289)
Supplement: Supplementary file 1 [file DataSheet1.DOCX]

**Table S1** Genes differentially expressed in *P. fluorescens* Pf0-1 in response to volatiles produced by *Collimonas pratensis* Ter 91.

| **SEQ_ID** | **Gene description** |
| --- | --- |
| \| Pfl_0362 \| \| --- \| \| Pfl_0553 \| \| Pfl_0650 \| \| Pfl_0673 \| \| Pfl_0685 \| \| Pfl_0686 \| \| Pfl_0729 \| \| Pfl_0973 \| \| Pfl_1152 \| \| Pfl_1153 \| \| Pfl_1167 \| \| Pfl_1168 \| \| Pfl_1169 \| \| Pfl_1170 \| \| Pfl_1247 \| \| Pfl_1250 \| \| Pfl_1507 \| \| Pfl_1527 \| \| Pfl_1528 \| \| Pfl_1566 \| \| Pfl_1567 \| \| Pfl_1606 \| \| Pfl_1640 \| \| Pfl_1746 \| \| Pfl_1812 \| \| Pfl_1830 \| \| Pfl_1918 \| \| Pfl_1971 \| \| Pfl_2128 \| \| Pfl_2129 \| \| Pfl_2585 \| \| Pfl_2662 \| \| Pfl_2663 \| \| Pfl_2664 \| \| Pfl_3051 \| \| Pfl_3052 \| \| Pfl_3190 \| \| Pfl_3441 \| \| Pfl_3442 \| \| Pfl_3463 \| \| Pfl_3464 \| \| Pfl_3465 \| \| Pfl_3954 \| \| Pfl_3992 \| \| Pfl_4022 \| \| Pfl_4036 \| \| Pfl_4308 \| \| Pfl_4391 \| \| Pfl_4392 \| \| Pfl_4419 \| \| Pfl_4431 \| \| Pfl_4518 \| \| Pfl_4654 \| \| Pfl_4708 \| \| Pfl_4715 \| \| Pfl_4734 \| \| Pfl_4735 \| \| Pfl_4758 \| \| Pfl_4794 \| \| Pfl_5043 \| \| Pfl_5171 \| \| Pfl_5230 \| \| Pfl_5239 \| \| Pfl_5694 \| \| Pfl_5695 \| | *Up-regulated genes with > 2-fold change*     \| Substrate-binding region of ABC-type glycine betaine transport system \| \| --- \| \| inner-membrane translocator \| \| Flp/Fap pilin component \| \| Basic membrane lipoprotein \| \| beta alanine-pyruvate transaminase \| \| Methylmalonate-semialdehyde dehydrogenase \| \| Phosphoglycerate/bisphosphoglycerate mutase \| \| extracellular solute-binding protein, family 3 \| \| hypothetical protein \| \| hypothetical protein \| \| hypothetical protein \| \| tail fiber protein, putative \| \| hypothetical protein \| \| Carbohydrate-binding, CenC-like \| \| inner-membrane translocator \| \| hypothetical protein \| \| Flagellin-like \| \| Flagellin-like \| \| Flagellar protein FlaG protein \| \| CheA Signal Transduction Histidine Kinases (STHK) \| \| chemotaxis-specific methylesterase \| \| Substrate-binding region of ABC-type glycine betaine transport system \| \| hypothetical protein \| \| hypothetical protein \| \| hypothetical protein \| \| hypothetical protein \| \| hypothetical protein \| \| hypothetical protein \| \| hypothetical protein \| \| hypothetical protein \| \| hypothetical protein \| \| hypothetical protein \| \| hypothetical protein \| \| GTP-binding protein, HSR1-like \| \| hypothetical protein \| \| 37kDa nucleoid-associated protein \| \| ATPase domain protein \| \| dihydropyrimidinase \| \| 4Fe-4S ferredoxin, iron-sulfur binding \| \| Dehydrogenase, E1 component \| \| Transketolase-like \| \| dihydrolipoamide acetyltransferase \| \| CheW protein \| \| Protein of unknown function DUF1127 \| \| chemotaxis sensory transducer \| \| hypothetical protein \| \| chemotaxis sensory transducer, Pas/Pac sensor \| \| glycine cleavage system protein H \| \| glycine dehydrogenase \| \| outer membrane porin \| \| chemotaxis sensory transducer, Cache sensor \| \| OmpA/MotB \| \| CheW protein \| \| Protein of unknown function DUF1302 \| \| chemotaxis sensory transducer \| \| Protein of unknown function DUF523 \| \| Transcriptional Regulator, MerR family \| \| chemotaxis sensory transducer \| \| Pentapeptide repeat \| \| hypothetical protein \| \| hypothetical protein \| \| Substrate-binding region of ABC-type glycine betaine transport system \| \| Binding-protein-dependent transport systems inner membrane component \| \| hypothetical protein \| \| hypothetical protein \| |
| \| Pfl_0043 \| \| --- \| \| Pfl_0097 \| \| Pfl_0100 \| \| Pfl_0101 \| \| Pfl_0105 \| \| Pfl_0110 \| \| Pfl_0193 \| \| Pfl_0194 \| \| Pfl_0223 \| \| Pfl_0256 \| \| Pfl_0445 \| \| Pfl_0567 \| \| Pfl_0654 \| \| Pfl_0655 \| \| Pfl_0748 \| \| Pfl_0877 \| \| Pfl_0949 \| \| Pfl_0950 \| \| Pfl_0951 \| \| Pfl_0952 \| \| Pfl_0953 \| \| Pfl_0954 \| \| Pfl_0955 \| \| Pfl_0956 \| \| Pfl_0957 \| \| Pfl_0958 \| \| Pfl_0959 \| \| Pfl_0960 \| \| Pfl_1031 \| \| Pfl_1066 \| \| Pfl_1067 \| \| Pfl_1200 \| \| Pfl_1212 \| \| Pfl_1324 \| \| Pfl_1412 \| \| Pfl_1440 \| \| Pfl_1442 \| \| Pfl_1482 \| \| Pfl_1629 \| \| Pfl_1639 \| \| Pfl_1657 \| \| Pfl_1727 \| \| Pfl_1733 \| \| Pfl_1802 \| \| Pfl_1807 \| \| Pfl_1839 \| \| Pfl_1840 \| \| Pfl_2029 \| \| Pfl_2095 \| \| Pfl_2097 \| \| Pfl_2107 \| \| Pfl_2108 \| \| Pfl_2109 \| \| Pfl_2110 \| \| Pfl_2403 \| \| Pfl_2404 \| \| Pfl_2531 \| \| Pfl_2534 \| \| Pfl_2535 \| \| Pfl_2538 \| \| Pfl_2544 \| \| Pfl_2546 \| \| Pfl_2547 \| \| Pfl_2565 \| \| Pfl_2568 \| \| Pfl_2572 \| \| Pfl_2613 \| \| Pfl_2614 \| \| Pfl_2615 \| \| Pfl_2621 \| \| Pfl_2647 \| \| Pfl_2648 \| \| Pfl_2650 \| \| Pfl_2652 \| \| Pfl_2653 \| \| Pfl_2692 \| \| Pfl_2695 \| \| Pfl_2723 \| \| Pfl_2732 \| \| Pfl_2786 \| \| Pfl_2788 \| \| Pfl_2789 \| \| Pfl_2791 \| \| Pfl_2792 \| \| Pfl_3112 \| \| Pfl_3132 \| \| Pfl_3292 \| \| Pfl_3324 \| \| Pfl_3326 \| \| Pfl_3509 \| \| Pfl_3517 \| \| Pfl_3539 \| \| Pfl_3570 \| \| Pfl_3580 \| \| Pfl_3701 \| \| Pfl_3702 \| \| Pfl_3706 \| \| Pfl_3717 \| \| Pfl_3730 \| \| Pfl_3753 \| \| Pfl_3762 \| \| Pfl_3763 \| \| Pfl_3799 \| \| Pfl_3890 \| \| Pfl_3969 \| \| Pfl_3970 \| \| Pfl_4093 \| \| Pfl_4100 \| \| Pfl_4101 \| \| Pfl_4102 \| \| Pfl_4107 \| \| Pfl_4132 \| \| Pfl_4133 \| \| Pfl_4134 \| \| Pfl_4214 \| \| Pfl_4267 \| \| Pfl_4268 \| \| Pfl_4351 \| \| Pfl_4357 \| \| Pfl_4432 \| \| Pfl_4433 \| \| Pfl_4579 \| \| Pfl_4774 \| \| Pfl_4969 \| \| Pfl_5035 \| \| Pfl_5036 \| \| Pfl_5037 \| \| Pfl_5039 \| \| Pfl_5040 \| \| Pfl_5243 \| \| Pfl_5353 \| \| Pfl_5504 \| \| Pfl_5542 \| \| Pfl_5619 \| \| Pfl_5625 \| | *Down-regulated genes with > 3-fold change*   \| hypothetical protein \| \| --- \| \| OsmC-like protein \| \| Protein of unknown function DUF1458 \| \| Protein of unknown function DUF883 \| \| hypothetical protein \| \| hypothetical protein \| \| hypothetical protein \| \| Thiosulphate-binding protein \| \| NLPA lipoprotein \| \| Taurine ABC transporter, periplasmic binding protein \| \| MscS Mechanosensitive ion channel \| \| Ferritin and Dps \| \| MaoC-like dehydratase \| \| 3-ketoacyl-(acyl-carrier-protein) reductase \| \| hypothetical protein \| \| sulfate adenylyltransferase subunit 2 \| \| Mannose-1-phosphate guanylyltransferase/mannose-6-phosphate isomerase \| \| alginate biosynthesis protein AlgF \| \| alginate biosynthesis protein AlgJ \| \| Membrane bound O-acyl transferase, MBOAT \| \| poly(beta-D-mannuronate) lyase \| \| alginate biosynthesis protein AlgX \| \| Carbohydrate binding and sugar hydrolysis \| \| outer membrane protein AlgE \| \| Sel1-like repeat \| \| alginate biosynthesis protein Alg44 \| \| alginate biosynthesis protein Alg8 \| \| UDP-glucose/GDP-mannose dehydrogenase \| \| hypothetical protein \| \| OmpA/MotB \| \| hypothetical protein \| \| hypothetical protein \| \| hypothetical protein \| \| hypothetical protein \| \| hypothetical protein \| \| hypothetical protein \| \| hypothetical protein \| \| hypothetical protein \| \| Amidohydrolase 2 \| \| L-sorbosone dehydrogenase \| \| hypothetical protein \| \| hypothetical protein \| \| hypothetical protein \| \| Zinc-containing alcohol dehydrogenase superfamily \| \| Glycoside hydrolase 15-like \| \| Short-chain dehydrogenase/reductase SDR \| \| hypothetical protein \| \| hypothetical protein \| \| DNA ligase \| \| Xanthine dehydrogenase \| \| Molybdopterin dehydrogenase, FAD-binding \| \| Ferredoxin \| \| hypothetical protein \| \| hypothetical protein \| \| Hemerythrin HHE cation binding region \| \| hypothetical protein \| \| Alpha amylase, catalytic region \| \| Alpha amylase, catalytic region \| \| Endonuclease/exonuclease/phosphatase \| \| Glycogen debranching enzyme GlgX \| \| (1,4)-alpha-D-glucan 1-alpha-D-glucosylmutase \| \| 4-alpha-glucanotransferase \| \| hypothetical protein \| \| Protein of unknown function DUF883 \| \| lipoprotein, putative \| \| conserved hypothetical protein \| \| hypothetical protein \| \| hypothetical protein \| \| hypothetical protein \| \| MCP methyltransferase, CheR-type \| \| CheB methylesterase \| \| Response regulator receiver domain protein (CheY) \| \| Histidine Kinase \| \| Possible circadian clock protein KaiC \| \| hypothetical protein \| \| Short-chain dehydrogenase/reductase SDR \| \| Protein of unknown function DUF1206 \| \| pyruvate decarboxylase \| \| Alpha/beta hydrolase fold \| \| Manganese containing catalase \| \| CinA-like \| \| hypothetical protein \| \| hypothetical protein \| \| Nitrite and sulphite reductase 4Fe-4S region \| \| hypothetical protein \| \| Polysaccharide deacetylase \| \| hypothetical protein \| \| chemotaxis sensory transducer \| \| hypothetical protein \| \| hypothetical protein \| \| hypothetical protein \| \| Hemerythrin HHE cation binding region \| \| Fructose-bisphosphate aldolase \| \| hypothetical protein \| \| hypothetical protein \| \| lipoprotein, putative \| \| Protein of unknown function UPF0118 \| \| hypothetical protein \| \| hypothetical protein \| \| hypothetical protein \| \| hypothetical protein \| \| hypothetical protein \| \| aconitate hydratase \| \| Vesicle-fusing ATPase \| \| hypothetical protein \| \| Fumarate hydratase, class II \| \| Asparagine synthase, glutamine-hydrolyzing \| \| GCN5-related N-acetyltransferase \| \| Peptidase M42 \| \| hypothetical protein \| \| hypothetical protein \| \| hypothetical protein \| \| hypothetical protein \| \| hypothetical protein \| \| hypothetical protein \| \| hypothetical protein \| \| CsbD-like \| \| hypothetical protein \| \| Glycosyl transferase, group 1 \| \| hypothetical protein \| \| Peptidase S16, ATP-dependent protease La \| \| hypothetical protein \| \| Rod shape-determining protein RodA \| \| Protein of unknown function DUF72 \| \| Aminoacyl-tRNA hydrolase \| \| Phospholipase D/Transphosphatidylase \| \| hypothetical protein \| \| hypothetical protein \| \| Betaine aldehyde dehydrogenase \| \| Peptidase C56, PfpI \| \| Protein of unknown function UPF0047 \| \| Phosphomannomutase \| \| hypothetical protein \| \| Aspartate ammonia-lyase \| |

**Table S2**  Genes differentially expressed in *P. fluorescens* Pf0-1 in response to volatiles produced by *Paenibacillus* sp. P4

| **SEQ_ID** | **Gene description** |
| --- | --- |
| \|  \| \| --- \| \| Pfl_0043 \| \| Pfl_0097 \| \| Pfl_0101 \| \| Pfl_0317 \| \| Pfl_0567 \| \| Pfl_0748 \| \| Pfl_0808 \| \| Pfl_0954 \| \| Pfl_0960 \| \| Pfl_1031 \| \| Pfl_1066 \| \| Pfl_1067 \| \| Pfl_1212 \| \| Pfl_1324 \| \| Pfl_1440 \| \| Pfl_1442 \| \| Pfl_1639 \| \| Pfl_1727 \| \| Pfl_1733 \| \| Pfl_2029 \| \| Pfl_2099 \| \| Pfl_2107 \| \| Pfl_2108 \| \| Pfl_2109 \| \| Pfl_2403 \| \| Pfl_2404 \| \| Pfl_2531 \| \| Pfl_2534 \| \| Pfl_2535 \| \| Pfl_2536 \| \| Pfl_2542 \| \| Pfl_2543 \| \| Pfl_2544 \| \| Pfl_2545 \| \| Pfl_2613 \| \| Pfl_2652 \| \| Pfl_2653 \| \| Pfl_2695 \| \| Pfl_2788 \| \| Pfl_3132 \| \| Pfl_3570 \| \| Pfl_3701 \| \| Pfl_3702 \| \| Pfl_3762 \| \| Pfl_4100 \| \| Pfl_4101 \| \| Pfl_4102 \| \| Pfl_4107 \| \| Pfl_4132 \| \| Pfl_4133 \| \| Pfl_4134 \| \| Pfl_4351 \| \| Pfl_4357 \| \| Pfl_4774 \| \| Pfl_5035 \| \| Pfl_5036 \| \| Pfl_5037 \| \| Pfl_5243 \| \|  \| \|  \| | *Up-regulated genes with > 2-fold change*   \|  \| \| --- \| \| hypothetical protein \| \| OsmC-like protein \| \| Protein of unknown function DUF883 \| \| Ferritin and Dps \| \| hypothetical protein \| \| hypothetical protein \| \| alginate biosynthesis protein AlgX \| \| UDP-glucose/GDP-mannose dehydrogenase \| \| hypothetical protein \| \| OmpA/MotB \| \| hypothetical protein \| \| hypothetical protein \| \| hypothetical protein \| \| hypothetical protein \| \| hypothetical protein \| \| L-sorbosone dehydrogenase \| \| hypothetical protein \| \| hypothetical protein \| \| hypothetical protein \| \| hypothetical protein \| \| Xanthine dehydrogenase \| \| Molybdopterin dehydrogenase, FAD-binding \| \| Ferredoxin \| \| hypothetical protein \| \| Hemerythrin HHE cation binding region \| \| hypothetical protein \| \| Alpha amylase, catalytic region \| \| Alpha amylase, catalytic region \| \| glycogen branching enzyme \| \| LmbE-like protein \| \| hypothetical protein \| \| Glycogen debranching enzyme GlgX \| \| hypothetical protein \| \| hypothetical protein  conserved hypothetical protein \| \| Histidine Kinase \| \| Possible circadian clock protein KaiC \| \| Short-chain dehydrogenase/reductase SDR \| \| Manganese containing catalase \| \| hypothetical protein \| \| Hemerythrin HHE cation binding region \| \| hypothetical protein \| \| hypothetical protein \| \| hypothetical protein \| \| Asparagine synthase, glutamine-hydrolyzing \| \| GCN5-related N-acetyltransferase \| \| Peptidase M42 \| \| hypothetical protein \| \| hypothetical protein \| \| hypothetical protein \| \| hypothetical protein \| \| CsbD-like \| \| hypothetical protein \| \| hypothetical protein \| \| Protein of unknown function DUF72 \| \| Aminoacyl-tRNA hydrolase \| \| Phospholipase D/Transphosphatidylase \| \| Betaine aldehyde dehydrogenase \| \|  \| \|  \| |
| \|  \| \| --- \| \| Pfl_0085 \| \| Pfl_0086 \| \| Pfl_0344 \| \| Pfl_0462 \| \| Pfl_0463 \| \| Pfl_0556 \| \| Pfl_0672 \| \| Pfl_0673 \| \| Pfl_0814 \| \| Pfl_1137 \| \| Pfl_1138 \| \| Pfl_1139 \| \| Pfl_1140 \| \| Pfl_1141 \| \| Pfl_1142 \| \| Pfl_1143 \| \| Pfl_1144 \| \| Pfl_1145 \| \| Pfl_1146 \| \| Pfl_1147 \| \| Pfl_1148 \| \| Pfl_1149 \| \| Pfl_1150 \| \| Pfl_1151 \| \| Pfl_1152 \| \| Pfl_1153 \| \| Pfl_1154 \| \| Pfl_1155 \| \| Pfl_1156 \| \| Pfl_1157 \| \| Pfl_1158 \| \| Pfl_1159 \| \| Pfl_1160 \| \| Pfl_1161 \| \| Pfl_1162 \| \| Pfl_1163 \| \| Pfl_1164 \| \| Pfl_1165 \| \| Pfl_1166 \| \| Pfl_1167 \| \| Pfl_1168 \| \| Pfl_1169 \| \| Pfl_1170 \| \| Pfl_1171 \| \| Pfl_1172 \| \| Pfl_1173 \| \| Pfl_1178 \| \| Pfl_1338 \| \| Pfl_1339 \| \| Pfl_1340 \| \| Pfl_1341 \| \| Pfl_1342 \| \| Pfl_1343 \| \| Pfl_1960 \| \| Pfl_1967 \| \| Pfl_2128 \| \| Pfl_2129 \| \| Pfl_2575 \| \| Pfl_2576 \| \| Pfl_3276 \| \| Pfl_3442 \| \| Pfl_4362 \| \| Pfl_4363 \| \| Pfl_4375 \| \| Pfl_4376 \| \| Pfl_4419 \| \| Pfl_5505 \| \|  \| | \| *Down-regulated genes with > 2-fold change* \| \| \| --- \| --- \| \| Glucose-methanol-choline oxidoreductase \| \| hypothetical protein \| \| Glutamine synthetase type I \| \| dihydrolipoamide acetyltransferase \| \| 2-oxo-acid dehydrogenase E1 component homodimeric type \| \| ABC transporter-like \| \| 2OG-Fe(II) oxygenase \| \| Basic membrane lipoprotein \| \| outer membrane porin \| \| pyocin R2_PP, holin \| \| hypothetical protein \| \| Phage baseplate assembly protein V \| \| hypothetical protein \| \| Baseplate J-like protein \| \| Phage tail protein I \| \| Phage Tail Collar \| \| hypothetical protein \| \| Phage tail sheath protein FI-like \| \| hypothetical protein \| \| hypothetical protein \| \| Phage tail tape measure protein TP901, core region \| \| Phage protein U-like \| \| Phage tail X \| \| Phage protein D-like \| \| hypothetical protein \| \| hypothetical protein \| \| Bacteriophage Mu tail sheath \| \| hypothetical protein \| \| Sigma-factor domain protein \| \| TMP \| \| DNA circulation-like \| \| Bacteriophage Mu P \| \| Phage baseplate assembly protein V \| \| Phage FluMu protein gp46 \| \| Baseplate J-like protein \| \| tail protein, putative \| \| hypothetical protein \| \| putative tail fiber assembly-like protein \| \| hypothetical protein \| \| hypothetical protein \| \| tail fiber protein, putative \| \| hypothetical protein \| \| Carbohydrate-binding, CenC-like \| \| hypothetical protein \| \| Pyocin R2_PP, lytic enzyme \| \| probable bacteriophage signal peptide protein \| \| hypothetical protein \| \| Allophanate hydrolase subunit 2 \| \| hypothetical protein \| \| hypothetical protein \| \| ABC transporter-like \| \| Binding-protein-dependent transport systems inner membrane component \| \| ABC transporter, periplasmic substrate-binding protein \| \| Transcriptional Regulator, AraC family \| \| Isochorismatase hydrolase \| \| hypothetical protein \| \| hypothetical protein \| \| extracellular solute-binding protein, family 1 \| \| agmatinase \| \| Aldehyde dehydrogenase \| \| 4Fe-4S ferredoxin, iron-sulfur binding \| \| 6-phosphogluconolactonase \| \| glucose-6-phosphate 1-dehydrogenase \| \| phosphogluconate dehydratase \| \| glyceraldehyde-3-phosphate dehydrogenase \| \| outer membrane porin \| \| Ammonium transporter \| |

**Table S3** Genes differentially expressed in *P. fluorescens* Pf0-1 in response to volatiles produced by *Serratia plymuthica* PRI-2C

| **SEQ_ID** | **Gene description** |
| --- | --- |
| \| \|  \| \| --- \| \| Pfl_0077 \| \| Pfl_0079 \| \| Pfl_0113 \| \| Pfl_0114 \| \| Pfl_0453 \| \| Pfl_0556 \| \| Pfl_0665 \| \| Pfl_0753 \| \| Pfl_0963 \| \| Pfl_1049 \| \| Pfl_1070 \| \| Pfl_1261 \| \| Pfl_1311 \| \| Pfl_1327 \| \| Pfl_1329 \| \| Pfl_1334 \| \| **Pfl_1507** \| \| Pfl_1515 \| \| Pfl_1516 \| \| **Pfl_1606** \| \| Pfl_1607 \| \| Pfl_1608 \| \| **Pfl_1640** \| \| Pfl_1718 \| \| Pfl_1739 \| \| Pfl_1811 \| \| Pfl_1817 \| \| Pfl_1926 \| \| **Pfl_1971** \| \| Pfl_2035 \| \| Pfl_2087 \| \| **Pfl_2128** \| \| Pfl_2250 \| \| Pfl_2251 \| \| Pfl_2260 \| \| Pfl_2347 \| \| Pfl_2601 \| \| **Pfl_2662** \| \| Pfl_2808 \| \| Pfl_2906 \| \| Pfl_2939 \| \| Pfl_3003 \| \| **Pfl_3190** \| \| Pfl_3221 \| \| Pfl_3326 \| \| Pfl_3341 \| \| Pfl_3393 \| \| Pfl_3421 \| \| Pfl_3439 \| \| **Pfl_3441** \| \| **Pfl_3442** \| \| **Pfl_3463** \| \| Pfl_3544 \| \| Pfl_3704 \| \| Pfl_3819 \| \| Pfl_3820 \| \| **Pfl_3992** \| \| **Pfl_4022** \| \| Pfl_4034 \| \| Pfl_4293 \| \| Pfl_4294 \| \| **Pfl_4308** \| \| Pfl_4385 \| \| Pfl_4386 \| \| Pfl_4387 \| \| **Pfl_4419** \| \| Pfl_4531 \| \| Pfl_4532 \| \| Pfl_4534 \| \| Pfl_4658 \| \| **Pfl_4734** \| \| **Pfl_4735** \| \| **Pfl_4794** \| \| Pfl_4825 \| \| Pfl_4862 \| \| Pfl_4982 \| \| Pfl_5185 \| \| **Pfl_5230** \| \| Pfl_5238 \| \| **Pfl_5239** \| \| Pfl_5261 \| \| Pfl_5317 \| \| Pfl_5417 \| \| Pfl_5441 \| \| **Pfl_5695** \| \| Pfl_5705 \| \|  \| \|  \| \| \| --- \| --- \| --- \| --- \| --- \| --- \| --- \| --- \| --- \| --- \| --- \| --- \| --- \| --- \| --- \| --- \| --- \| --- \| --- \| --- \| --- \| --- \| --- \| --- \| --- \| --- \| --- \| --- \| --- \| --- \| --- \| --- \| --- \| --- \| --- \| --- \| --- \| --- \| --- \| --- \| --- \| --- \| --- \| --- \| --- \| --- \| --- \| --- \| --- \| --- \| --- \| --- \| --- \| --- \| --- \| --- \| --- \| --- \| --- \| --- \| --- \| --- \| --- \| --- \| --- \| --- \| --- \| --- \| --- \| --- \| --- \| --- \| --- \| --- \| --- \| --- \| --- \| --- \| --- \| --- \| --- \| --- \| --- \| --- \| --- \| --- \| --- \| --- \| --- \| --- \| \|  \| | *Up-regulated genes with > 2-fold change*   \| \| Cytochrome C oxidase assembly protein \| \| --- \| \| Cytochrome c oxidase, subunit II \| \| NAD/NADP transhydrogenase, membrane-spanning dIIa subunit \| \| NAD(P)(+) transhydrogenase (AB-specific) \| \| Sodium/proline symporter \| \| ABC transporter-like \| \| Hypothetical protein \| \| L-lactate permease \| \| PhoH-like protein \| \| Betaine-aldehyde dehydrogenase \| \| Adenylate kinase \| \| Hypothetical protein \| \| Iron-containing alcohol dehydrogenase \| \| Trans-aconitate methyltransferase, putative \| \| Hypothetical protein \| \| Alpha/beta hydrolase fold \| \| **Flagellin-like** \| \| Transferase hexapeptide repeat \| \| Cephalosporin hydroxylase \| \| **Substrate-binding region of ABC-type glycine betaine transport system** \| \| Protein of unknown function DUF485 \| \| Acetate permease \| \| **Hypothetical protein** \| \| Isochorismatase hydrolase \| \| Acetyl-CoA acetyltransferase \| \| Transcriptional Regulator, Crp/Fnr family \| \| Hypothetical protein \| \| Hypothetical protein \| \| **Hypothetical protein** \| \| Hypothetical protein \| \| Hypothetical protein \| \| **Hypothetical protein** \| \| Response regulator receiver domain protein (CheY) \| \| PAS/PAC Sensor Signal Transduction Histidine Kinase \| \| Creatininase \| \| Zinc-containing alcohol dehydrogenase superfamily \| \| Chemotaxis sensory transducer \| \| **Hypothetical protein** \| \| Lipoprotein, putative \| \| Hypothetical protein \| \| Zinc-containing alcohol dehydrogenase superfamily \| \| Hypothetical protein \| \| **ATPase domain protein** \| \| Hypothetical protein \| \| Chemotaxis sensory transducer \| \| GCN5-related N-acetyltransferase \| \| UspA \| \| Hypothetical protein \| \| N-carbamoyl-L-amino acid amidohydrolase \| \| **Dihydropyrimidinase** \| \| **4Fe-4S ferredoxin, iron-sulfur binding** \| \| **Dehydrogenase, E1 component** \| \| Chorismate mutase \| \| Hypothetical protein \| \| Hypothetical protein \| \| Hypothetical protein \| \| **Protein of unknown function DUF1127** \| \| **Chemotaxis sensory transducer** \| \| Amino acid permease-associated region \| \| Scetyl-coenzyme A synthetase \| \| Hypothetical protein \| \| **Chemotaxis sensory transducer, Pas/Pac sensor** \| \| Arginine/ornithine antiporter \| \| Arginine deiminase \| \| Ornithine carbamoyltransferase \| \| **Outer membrane porin** \| \| Aquaporin \| \| Glycerol kinase \| \| FAD dependent oxidoreductase \| \| Chemotaxis sensory transducer, Pas/Pac sensor \| \| **Protein of unknown function DUF523** \| \| **Transcriptional Regulator, MerR family** \| \| **Pentapeptide repeat** \| \| Hypothetical protein \| \| PhnA protein \| \| MaoC-like dehydratase \| \| Cyclic nucleotide-binding (cNMP-BD) protein \| \| **Substrate-binding region of ABC-type glycine betaine transport system** \| \| Substrate-binding region of ABC-type glycine betaine transport system \| \| **Binding-protein-dependent transport systems inner membrane component** \| \| Hypothetical protein \| \| NLP/P60 \| \| Outer membrane porin \| \| Secretion protein HlyD \| \| **Hypothetical protein** \| \| Hypothetical protein \| \|  \| \|  \| \| \| --- \| --- \| --- \| --- \| --- \| --- \| --- \| --- \| --- \| --- \| --- \| --- \| --- \| --- \| --- \| --- \| --- \| --- \| --- \| --- \| --- \| --- \| --- \| --- \| --- \| --- \| --- \| --- \| --- \| --- \| --- \| --- \| --- \| --- \| --- \| --- \| --- \| --- \| --- \| --- \| --- \| --- \| --- \| --- \| --- \| --- \| --- \| --- \| --- \| --- \| --- \| --- \| --- \| --- \| --- \| --- \| --- \| --- \| --- \| --- \| --- \| --- \| --- \| --- \| --- \| --- \| --- \| --- \| --- \| --- \| --- \| --- \| --- \| --- \| --- \| --- \| --- \| --- \| --- \| --- \| --- \| --- \| --- \| --- \| --- \| --- \| --- \| --- \| --- \| |
| \|  \| \| --- \| \| Pfl_0039 \| \| **Pfl_0043** \| \| Pfl_0087 \| \| **Pfl_0097** \| \| Pfl_0098 \| \| **Pfl_0101** \| \| Pfl_0106 \| \| **Pfl_0193** \| \| **Pfl_0194** \| \| **Pfl_0223** \| \| **Pfl_0256** \| \| Pfl_0264 \| \| Pfl_0352 \| \| Pfl_0360 \| \| Pfl_0361 \| \| Pfl_0363 \| \| Pfl_0364 \| \| Pfl_0374 \| \| Pfl_0438 \| \| **Pfl_0445** \| \| Pfl_0625 \| \| Pfl_0627 \| \| Pfl_0685 \| \| Pfl_0729 \| \| Pfl_0730 \| \| **Pfl_0748** \| \| **Pfl_0877** \| \| **Pfl_0949** \| \| **Pfl_0950** \| \| **Pfl_0951** \| \| **Pfl_0952** \| \| **Pfl_0953** \| \| **Pfl_0954** \| \| **Pfl_0955** \| \| **Pfl_0956** \| \| **Pfl_0957** \| \| **Pfl_0958** \| \| **Pfl_0959** \| \| **Pfl_0960** \| \| Pfl_1031 \| \| Pfl_1038 \| \| **Pfl_1066** \| \| **Pfl_1067** \| \| Pfl_1089 \| \| Pfl_1105 \| \| **Pfl_1212** \| \| Pfl_1214 \| \| Pfl_1226 \| \| Pfl_1229 \| \| **Pfl_1324** \| \| Pfl_1356 \| \| Pfl_1365 \| \| Pfl_1387 \| \| Pfl_1428 \| \| **Pfl_1440** \| \| **Pfl_1482** \| \| Pfl_1586 \| \| Pfl_1602 \| \| Pfl_1603 \| \| Pfl_1628 \| \| **Pfl_1639** \| \| **Pfl_1727** \| \| **Pfl_1733** \| \| Pfl_1742 \| \| Pfl_1770 \| \| Pfl_1784 \| \| Pfl_1785 \| \| **Pfl_1802** \| \| Pfl_1902 \| \| Pfl_1930 \| \| **Pfl_2029** \| \| **Pfl_2095** \| \| **Pfl_2097** \| \| **Pfl_2107** \| \| **Pfl_2108** \| \| **Pfl_2109** \| \| Pfl_2162 \| \| Pfl_2190 \| \| Pfl_2289 \| \| Pfl_2376 \| \| **Pfl_2403** \| \| **Pfl_2404** \| \| Pfl_2508 \| \| Pfl_2509 \| \| **Pfl_2531** \| \| **Pfl_2535** \| \| **Pfl_2538** \| \| **Pfl_2544** \| \| **Pfl_2547** \| \| Pfl_2562 \| \| **Pfl_2565** \| \| **Pfl_2568** \| \| Pfl_2584 \| \| Pfl_2587 \| \| **Pfl_2621** \| \| **Pfl_2647** \| \| **Pfl_2648** \| \| Pfl_2649 \| \| **Pfl_2650** \| \| **Pfl_2652** \| \| **Pfl_2653** \| \| **Pfl_2692** \| \| Pfl_2698 \| \| Pfl_2699 \| \| **Pfl_2732** \| \| **Pfl_2788** \| \| Pfl_2799 \| \| Pfl_2827 \| \| Pfl_2834 \| \| Pfl_2842 \| \| Pfl_2843 \| \| Pfl_2844 \| \| Pfl_2848 \| \| Pfl_2852 \| \| Pfl_2853 \| \| Pfl_2854 \| \| Pfl_2966 \| \| Pfl_2967 \| \| **Pfl_3112** \| \| Pfl_3113 \| \| **Pfl_3132** \| \| Pfl_3134 \| \| Pfl_3136 \| \| Pfl_3137 \| \| Pfl_3276 \| \| **Pfl_3324** \| \| Pfl_3445 \| \| **Pfl_3509** \| \| **Pfl_3517** \| \| **Pfl_3570** \| \| **Pfl_3580** \| \| Pfl_3648 \| \| **Pfl_3701** \| \| **Pfl_3702** \| \| **Pfl_3706** \| \| **Pfl_3717** \| \| **Pfl_3730** \| \| **Pfl_3753** \| \| **Pfl_3762** \| \| **Pfl_3763** \| \| **Pfl_3799** \| \| Pfl_3847 \| \| Pfl_3858 \| \| Pfl_3859 \| \| Pfl_3958 \| \| **Pfl_3969** \| \| **Pfl_3970** \| \| Pfl_3971 \| \| Pfl_4069 \| \| Pfl_4070 \| \| **Pfl_4100** \| \| **Pfl_4101** \| \| **Pfl_4107** \| \| **Pfl_4132** \| \| **Pfl_4133** \| \| **Pfl_4134** \| \| **Pfl_4214** \| \| Pfl_4239 \| \| **Pfl_4267** \| \| **Pfl_4268** \| \| Pfl_4336 \| \| Pfl_4349 \| \| **Pfl_4351** \| \| **Pfl_4357** \| \| Pfl_4499 \| \| Pfl_4500 \| \| **Pfl_4579** \| \| Pfl_4607 \| \| Pfl_4609 \| \| Pfl_4611 \| \| Pfl_4621 \| \| Pfl_4668 \| \| Pfl_4669 \| \| Pfl_4670 \| \| Pfl_4673 \| \| Pfl_4677 \| \| Pfl_4678 \| \| Pfl_4679 \| \| Pfl_4680 \| \| Pfl_4681 \| \| Pfl_4682 \| \| **Pfl_4774** \| \| Pfl_4872 \| \| Pfl_4873 \| \| Pfl_4926 \| \| **Pfl_5035** \| \| **Pfl_5036** \| \| **Pfl_5037** \| \| **Pfl_5039** \| \| **Pfl_5040** \| \| Pfl_5051 \| \| **Pfl_5243** \| \| Pfl_5265 \| \| Pfl_5336 \| \| **Pfl_5353** \| \| **Pfl_5542** \| \| Pfl_5543 \| \| Pfl_5670 \| | \| *Down-regulated genes with > 2-fold change* \| \| --- \| \| \|  \| \| --- \| \| hypothetical protein \| \| **hypothetical protein** \| \| Protein tyrosine/serine phosphatase \| \| **OsmC-like protein** \| \| luciferase-like \| \| **Protein of unknown function DUF883** \| \| hypothetical protein \| \| **hypothetical protein** \| \| **Thiosulphate-binding protein** \| \| **NLPA lipoprotein** \| \| **Taurine ABC transporter, periplasmic binding protein** \| \| Putative diguanylate phosphodiesterase (EAL domain) \| \| Inositol phosphatase/fructose-1,6-bisphosphatase \| \| urocanate hydratase \| \| Permease for cytosine/purines, uracil, thiamine, allantoin \| \| Binding-protein-dependent transport systems inner membrane component \| \| ABC transporter-like \| \| glucosyltransferase MdoH \| \| acyl carrier protein \| \| **MscS Mechanosensitive ion channel** \| \| NUDIX hydrolase \| \| arginine decarboxylase \| \| beta alanine-pyruvate transaminase \| \| Phosphoglycerate/bisphosphoglycerate mutase \| \| PAP2 superfamily protein \| \| **hypothetical protein** \| \| **sulfate adenylyltransferase subunit 2** \| \| **Mannose-1-phosphate guanylyltransferase/mannose-6-phosphate isomerase** \| \| **alginate biosynthesis protein AlgF** \| \| **alginate biosynthesis protein AlgJ** \| \| **Membrane bound O-acyl transferase, MBOAT** \| \| **poly(beta-D-mannuronate) lyase** \| \| **alginate biosynthesis protein AlgX** \| \| **Carbohydrate binding and sugar hydrolysis** \| \| **outer membrane protein AlgE** \| \| **Sel1-like repeat** \| \| **alginate biosynthesis protein Alg44** \| \| **alginate biosynthesis protein Alg8** \| \| **UDP-glucose/GDP-mannose dehydrogenase** \| \| hypothetical protein \| \| Twin-arginine translocation pathway signal \| \| **OmpA/MotB** \| \| **hypothetical protein** \| \| Glycosyl transferase, family 2 \| \| Di-trans-poly-cis-decaprenylcistransferase \| \| **hypothetical protein** \| \| Glutathione S-transferase-like \| \| hydrolase, putative \| \| pyridoxamine 5'-phosphate oxidase \| \| **hypothetical protein** \| \| Predicted signal transduction protein \| \| Peptidase S1C, Do \| \| hypothetical protein \| \| Peptidyl-prolyl cis-trans isomerase, cyclophilin type \| \| **hypothetical protein** \| \| **hypothetical protein** \| \| hypothetical protein \| \| VacJ-like lipoprotein \| \| hypothetical protein \| \| OsmC-like protein \| \| **L-sorbosone dehydrogenase** \| \| **hypothetical protein** \| \| **hypothetical protein** \| \| Mg2+ transporter protein, CorA-like \| \| phosphoenolpyruvate synthase \| \| Response regulator receiver (CheY) and ANTAR domain protein \| \| nitrate-binding protein NasS, putative \| \| **hypothetical protein** \| \| amidophosphoribosyltransferase \| \| hypothetical protein \| \| **hypothetical protein** \| \| **hypothetical protein** \| \| **DNA ligase** \| \| **Xanthine dehydrogenase** \| \| **Molybdopterin dehydrogenase, FAD-binding** \| \| **Ferredoxin** \| \| Multi-sensor Signal Transduction Histidine Kinase \| \| Transcriptional Regulator, LysR family \| \| hypothetical protein \| \| Fe2+-dicitrate sensor membrane component-like \| \| **hypothetical protein** \| \| **Hemerythrin HHE cation binding region** \| \| hypothetical protein \| \| hypothetical protein \| \| **hypothetical protein** \| \| **Alpha amylase, catalytic region** \| \| **Endonuclease/exonuclease/phosphatase** \| \| **Glycogen debranching enzyme GlgX** \| \| **4-alpha-glucanotransferase** \| \| hypothetical protein \| \| **hypothetical protein** \| \| **Protein of unknown function DUF883** \| \| Sulfatase \| \| Glucose-6-phosphate dehydrogenase \| \| **hypothetical protein** \| \| **MCP methyltransferase, CheR-type** \| \| **CheB methylesterase** \| \| Response Regulator Receiver Signal Transduction Histidine Kinase \| \| **Response regulator receiver domain protein (CheY)** \| \| **Histidine Kinase** \| \| **Possible circadian clock protein KaiC** \| \| **hypothetical protein** \| \| Formate dehydrogenase, alpha subunit, anaerobic \| \| Formate dehydrogenase, beta subunit \| \| **pyruvate decarboxylase** \| \| **Manganese containing catalase** \| \| Transcriptional Regulator, GntR family \| \| Transcriptional Regulator, AraC family \| \| Phosphoglycerate/bisphosphoglycerate mutase \| \| Glycosyl transferase, family 2 \| \| hypothetical protein \| \| Polysaccharide deacetylase \| \| UDP-glucose 6-dehydrogenase \| \| argininosuccinate lyase \| \| Amino acid ABC transporter, permease protein, 3-TM region, His/Glu/Gln/Arg/opine \| \| ABC transporter-like \| \| benzoate 1,2-dioxygenase ferredoxin reductase subunit \| \| Aromatic-ring-hydroxylating dioxygenase, beta subunit \| \| **Nitrite and sulphite reductase 4Fe-4S region** \| \| Uncharacterized conserved protein UCP030820 \| \| **hypothetical protein** \| \| Anti-Sigma-factor antagonist (STAS) \| \| Anti-Sigma-regulatory factor (Ser/Thr protein kinase and phosphatase) \| \| Histidine Kinase \| \| Aldehyde dehydrogenase \| \| **hypothetical protein** \| \| GCN5-related N-acetyltransferase \| \| **hypothetical protein** \| \| **hypothetical protein** \| \| **Hemerythrin HHE cation binding region** \| \| **Fructose-bisphosphate aldolase** \| \| acetyltransferase, GNAT family \| \| **hypothetical protein** \| \| **hypothetical protein** \| \| **lipoprotein, putative** \| \| **Protein of unknown function UPF0118** \| \| **hypothetical protein** \| \| **hypothetical protein** \| \| **hypothetical protein** \| \| **hypothetical protein** \| \| **hypothetical protein** \| \| transaldolase \| \| Lipoprotein releasing system, ATP-binding protein \| \| Lipoprotein releasing system, transmembrane protein, LolC/E family \| \| hypothetical protein \| \| **Vesicle-fusing ATPase** \| \| **hypothetical protein** \| \| hypothetical protein \| \| Glycosyltransferase-like \| \| UDP-glucose 6-dehydrogenase \| \| **Asparagine synthase, glutamine-hydrolyzing** \| \| **GCN5-related N-acetyltransferase** \| \| **hypothetical protein** \| \| **hypothetical protein** \| \| **hypothetical protein** \| \| **hypothetical protein** \| \| **hypothetical protein** \| \| Periplasmic Sensor Signal Transduction Histidine Kinase \| \| **hypothetical protein** \| \| **hypothetical protein** \| \| Carbohydrate kinase, thermoresistant glucokinase \| \| peptide deformylase \| \| **CsbD-like** \| \| **hypothetical protein** \| \| Sulfatase \| \| Phosphoesterase, PA-phosphatase-like \| \| **Peptidase S16, ATP-dependent protease La** \| \| Ferredoxin, 2Fe-2S type \| \| co-chaperone HscB \| \| FeS cluster assembly scaffold IscU \| \| queuine tRNA-ribosyltransferase \| \| cell division protein FtsZ \| \| Cell division protein FtsA \| \| Cell division protein FtsQ \| \| N-acetylglucosaminyl transferase \| \| UDP-N-acetylmuramoylalanyl-D-glutamyl-2, 6-diaminopimelate-D-alanyl-D-alanyl ligase \| \| UDP-N-acetylmuramoylalanyl-D-glutamate--2, 6-diaminopimelate ligase \| \| Penicillin-binding protein, transpeptidase \| \| Cell division protein, FtsL-like \| \| S-adenosyl-methyltransferase \| \| hypothetical protein \| \| **hypothetical protein** \| \| lipoprotein, putative \| \| GreA/GreB family elongation factor \| \| Predicted periplasmic ligand-binding sensor protein \| \| **Protein of unknown function DUF72** \| \| **Aminoacyl-tRNA hydrolase** \| \| **Phospholipase D/Transphosphatidylase** \| \| **hypothetical protein** \| \| **hypothetical protein** \| \| excinuclease ABC subunit A \| \| **Betaine aldehyde dehydrogenase** \| \| phosphoglycerate kinase \| \| ABC transporter-like \| \| **Peptidase C56, PfpI** \| \| **Phosphomannomutase** \| \| acetylglutamate kinase \| \| Band 7 protein \| \| |

Differently expressed genes marked in bold are similar with genes differentially expressed in response to volatiles by *C. pratensis* listed in Table 1

**Table S4**  Genes differentially expressed in *P. fluorescens* Pf0-1 in response to volatiles produced by *Pedobacter* sp. V48.

| **SEQ_ID** | **Gene description** |
| --- | --- |
| \| \|  \| \| --- \| \| Pfl_0101 \| \| Pfl_0110 \| \| Pfl_0567 \| \| Pfl_0748 \| \| Pfl_0960 \| \| Pfl_1066 \| \| Pfl_1067 \| \| Pfl_1338 \| \| Pfl_1339 \| \| Pfl_1340 \| \| Pfl_1341 \| \| Pfl_1342 \| \| Pfl_1343 \| \| Pfl_1727 \| \| Pfl_1746 \| \| Pfl_1778 \| \| Pfl_1782 \| \| Pfl_1783 \| \| Pfl_1785 \| \| Pfl_2029 \| \| Pfl_2107 \| \| Pfl_2403 \| \| Pfl_2404 \| \| Pfl_2531 \| \| Pfl_2544 \| \| Pfl_2565 \| \| Pfl_2576 \| \| Pfl_2613 \| \| Pfl_2907 \| \| Pfl_3132 \| \| Pfl_3570 \| \| Pfl_3701 \| \| Pfl_3702 \| \| Pfl_3762 \| \| Pfl_3820 \| \| Pfl_4101 \| \| Pfl_4107 \| \| Pfl_4132 \| \| Pfl_4133 \| \| Pfl_4351 \| \| Pfl_4357 \| \| Pfl_5035 \| \| Pfl_5036 \| \| \| --- \| --- \| --- \| --- \| --- \| --- \| --- \| --- \| --- \| --- \| --- \| --- \| --- \| --- \| --- \| --- \| --- \| --- \| --- \| --- \| --- \| --- \| --- \| --- \| --- \| --- \| --- \| --- \| --- \| --- \| --- \| --- \| --- \| --- \| --- \| --- \| --- \| --- \| --- \| --- \| --- \| --- \| --- \| --- \| --- \| | *Up-regulated genes with > 2-fold change*   \| \|  \| \| --- \| \| Protein of unknown function DUF883 \| \| hypothetical protein \| \| Ferritin and Dps \| \| hypothetical protein \| \| UDP-glucose/GDP-mannose dehydrogenase \| \| OmpA/MotB \| \| hypothetical protein \| \| Allophanate hydrolase subunit 2 \| \| hypothetical protein \| \| hypothetical protein \| \| ABC transporter-like \| \| Binding-protein-dependent transport systems inner membrane component \| \| ABC transporter, periplasmic substrate-binding protein \| \| hypothetical protein \| \| hypothetical protein \| \| Uroporphyrin-III C-methyltransferase-like \| \| Protein serine/threonine phosphatase \| \| Nitrate transporter \| \| nitrate-binding protein NasS, putative \| \| hypothetical protein \| \| Xanthine dehydrogenase \| \| hypothetical protein \| \| Hemerythrin HHE cation binding region \| \| hypothetical protein \| \| Glycogen debranching enzyme GlgX \| \| hypothetical protein \| \| agmatinase \| \| conserved hypothetical protein \| \| chemotaxis sensory transducer \| \| hypothetical protein \| \| Hemerythrin HHE cation binding region \| \| hypothetical protein \| \| hypothetical protein \| \| hypothetical protein \| \| hypothetical protein \| \| GCN5-related N-acetyltransferase \| \| hypothetical protein \| \| hypothetical protein \| \| hypothetical protein \| \| CsbD-like \| \| hypothetical protein \| \| Protein of unknown function DUF72 \| \| Aminoacyl-tRNA hydrolase \| \| \| --- \| --- \| --- \| --- \| --- \| --- \| --- \| --- \| --- \| --- \| --- \| --- \| --- \| --- \| --- \| --- \| --- \| --- \| --- \| --- \| --- \| --- \| --- \| --- \| --- \| --- \| --- \| --- \| --- \| --- \| --- \| --- \| --- \| --- \| --- \| --- \| --- \| --- \| --- \| --- \| --- \| --- \| --- \| --- \| --- \| \|  \| |
| \|  \| \| --- \| \| Pfl_0077 \| \| Pfl_1482 \| \| Pfl_1518 \| \| Pfl_2021 \| \| Pfl_2575 \| \| Pfl_2903 \| \| Pfl_3464 \| \| Pfl_3465 \| \| Pfl_5629 \| | *Down-regulated genes with > 2-fold change*   \|  \| \| --- \| \| cytochrome C oxidase assembly protein \| \| hypothetical protein \| \| NAD-dependent epimerase/dehydratase \| \| Polysaccharide export protein \| \| extracellular solute-binding protein, family 1 \| \| General substrate transporter \| \| Transketolase-like \| \| dihydrolipoamide acetyltransferase \| \| hypothetical protein \| |

**Table S5** Genes differentially expressed in *P. fluorescens* Pf0-1 in response to volatiles produced by the mix of all four bacteria.

| **SEQ_ID** | **Gene description** |
| --- | --- |
| \|  \| \| --- \| \| \| Pfl_0175 \| \| --- \| \| Pfl_0910 \| \| Pfl_1049 \| \| Pfl_1311 \| \| Pfl_1746 \| \| Pfl_2403 \| \| Pfl_3367 \| \| Pfl_3516 \| \| Pfl_3630 \| \| Pfl_3768 \| \| Pfl_3819 \| \| Pfl_3846 \| \| Pfl_4332 \| \| Pfl_4351 \| \| Pfl_4431 \| \| Pfl_4532 \| \| Pfl_4534 \| \| Pfl_5441 \| \| Pfl_5600 \| \| | *Up-regulated genes with > 2-fold change*   \| \| hypothetical protein \| \| --- \| \| homogentisate 1,2-dioxygenase \| \| Betaine-aldehyde dehydrogenase \| \| Iron-containing alcohol dehydrogenase \| \| hypothetical protein \| \| hypothetical protein \| \| hypothetical protein \| \| Ferredoxin \| \| extracellular solute-binding protein, family 1 \| \| chemotaxis sensory transducer \| \| hypothetical protein \| \| hypothetical protein \| \| transcription activator, effector binding \| \| CsbD-like \| \| chemotaxis sensory transducer, Cache sensor \| \| glycerol kinase \| \| FAD dependent oxidoreductase \| \| Secretion protein HlyD \| \| Histone-like DNA-binding protein \| \| \| --- \| --- \| --- \| --- \| --- \| --- \| --- \| --- \| --- \| --- \| --- \| --- \| --- \| --- \| --- \| --- \| --- \| --- \| --- \| --- \| |
| \|  \| \| --- \| \| Pfl_0085 \| \| Pfl_0086 \| \| Pfl_0339 \| \| Pfl_0340 \| \| Pfl_0344 \| \| Pfl_0462 \| \| Pfl_0463 \| \| Pfl_0561 \| \| Pfl_0586 \| \| Pfl_0590 \| \| Pfl_0671 \| \| Pfl_0672 \| \| Pfl_0673 \| \| Pfl_0893 \| \| Pfl_1136 \| \| Pfl_1140 \| \| Pfl_1141 \| \| Pfl_1142 \| \| Pfl_1145 \| \| Pfl_1147 \| \| Pfl_1149 \| \| Pfl_1151 \| \| Pfl_1152 \| \| Pfl_1160 \| \| Pfl_1167 \| \| Pfl_1168 \| \| Pfl_1169 \| \| Pfl_1170 \| \| Pfl_1171 \| \| Pfl_1173 \| \| Pfl_1178 \| \| Pfl_1334 \| \| Pfl_1338 \| \| Pfl_1339 \| \| Pfl_1340 \| \| Pfl_1341 \| \| Pfl_1342 \| \| Pfl_1343 \| \| Pfl_1735 \| \| Pfl_1736 \| \| Pfl_1737 \| \| Pfl_1960 \| \| Pfl_1966 \| \| Pfl_1967 \| \| Pfl_2575 \| \| Pfl_2576 \| \| Pfl_3439 \| \| Pfl_3442 \| \| Pfl_3443 \| \| Pfl_3997 \| \| Pfl_4294 \| \| Pfl_4322 \| \| Pfl_4362 \| \| Pfl_4363 \| \| Pfl_4375 \| \| Pfl_4376 \| \| Pfl_4459 \| \| Pfl_5406 \| \| Pfl_5407 \| \| Pfl_5459 \| \| Pfl_5460 \| \| Pfl_5461 \| \| Pfl_5462 \| \| Pfl_5505 \| \| Pfl_5506 \| | \| *Down-regulated genes with > 2-fold change* \| \| --- \| \| Glucose-methanol-choline oxidoreductase \| \| hypothetical protein \| \| Nitrogen Metabolism Transcriptional Regulator, NtrC, Fis Family \| \| Signal transduction histidine kinase, nitrogen specific, NtrB \| \| Glutamine synthetase type I \| \| dihydrolipoamide acetyltransferase \| \| 2-oxo-acid dehydrogenase E1 component homodimeric type \| \| Urease accessory protein UreG \| \| ABC transporter-like \| \| branched-chain amino acid ABC transporter, periplasmic amino acid-binding protein \| \| adenosine deaminase \| \| 2OG-Fe(II) oxygenase \| \| Basic membrane lipoprotein \| \| hypothetical protein \| \| hypothetical protein \| \| hypothetical protein \| \| Baseplate J-like protein \| \| Phage tail protein I \| \| Phage tail sheath protein FI-like \| \| hypothetical protein \| \| Phage protein U-like \| \| Phage protein D-like \| \| hypothetical protein \| \| Phage baseplate assembly protein V \| \| hypothetical protein \| \| tail fiber protein, putative \| \| hypothetical protein \| \| Carbohydrate-binding, CenC-like \| \| hypothetical protein \| \| probable bacteriophage signal peptide protein \| \| hypothetical protein \| \| Alpha/beta hydrolase fold \| \| Allophanate hydrolase subunit 2 \| \| hypothetical protein \| \| hypothetical protein \| \| ABC transporter-like \| \| Binding-protein-dependent transport systems inner membrane component \| \| ABC transporter, periplasmic substrate-binding protein \| \| hypothetical protein \| \| Protein of unknown function DUF403 \| \| Transglutaminase-like \| \| Transcriptional Regulator, AraC family \| \| hypothetical protein \| \| Isochorismatase hydrolase \| \| extracellular solute-binding protein, family 1 \| \| agmatinase \| \| N-carbamoyl-L-amino acid amidohydrolase \| \| 4Fe-4S ferredoxin, iron-sulfur binding \| \| dihydropyrimidine dehydrogenase \| \| OsmC-like protein \| \| hypothetical protein \| \| transmembrane pair \| \| 6-phosphogluconolactonase \| \| glucose-6-phosphate 1-dehydrogenase \| \| phosphogluconate dehydratase \| \| glyceraldehyde-3-phosphate dehydrogenase \| \| Citrate-proton symport \| \| hypothetical protein \| \| Glutamine synthetase, catalytic region \| \| extracellular solute-binding protein, family 3 \| \| Amino acid ABC transporter, permease protein, 3-TM region, His/Glu/Gln/Arg/opine \| \| Amino acid ABC transporter, permease protein, 3-TM region, His/Glu/Gln/Arg/opine \| \| ABC transporter-like \| \| Ammonium transporter \| \| Nitrogen regulatory protein P-II (GlnB, GlnK) \| |

The differentially expressed genes were identified using the false discovery rate (Benjamini-Hochberg) correction method with 99% confidence (P<0.05)

The common genes differentially expressed in *P. fluorescens* Pf0-1 in response to volatiles produced by 4 bacterial species and a mixture of these species are listed in Table 1 and excluded from these tables.
