# Supplementary figures and images for: Volatile-mediated interactions between phylogenetically different soil bacteria
Source: Front Microbiol. 2014 Jun 11;5:289. doi: 10.3389/fmicb.2014.00289 (PMC4052926; doi:10.3389/fmicb.2014.00289)

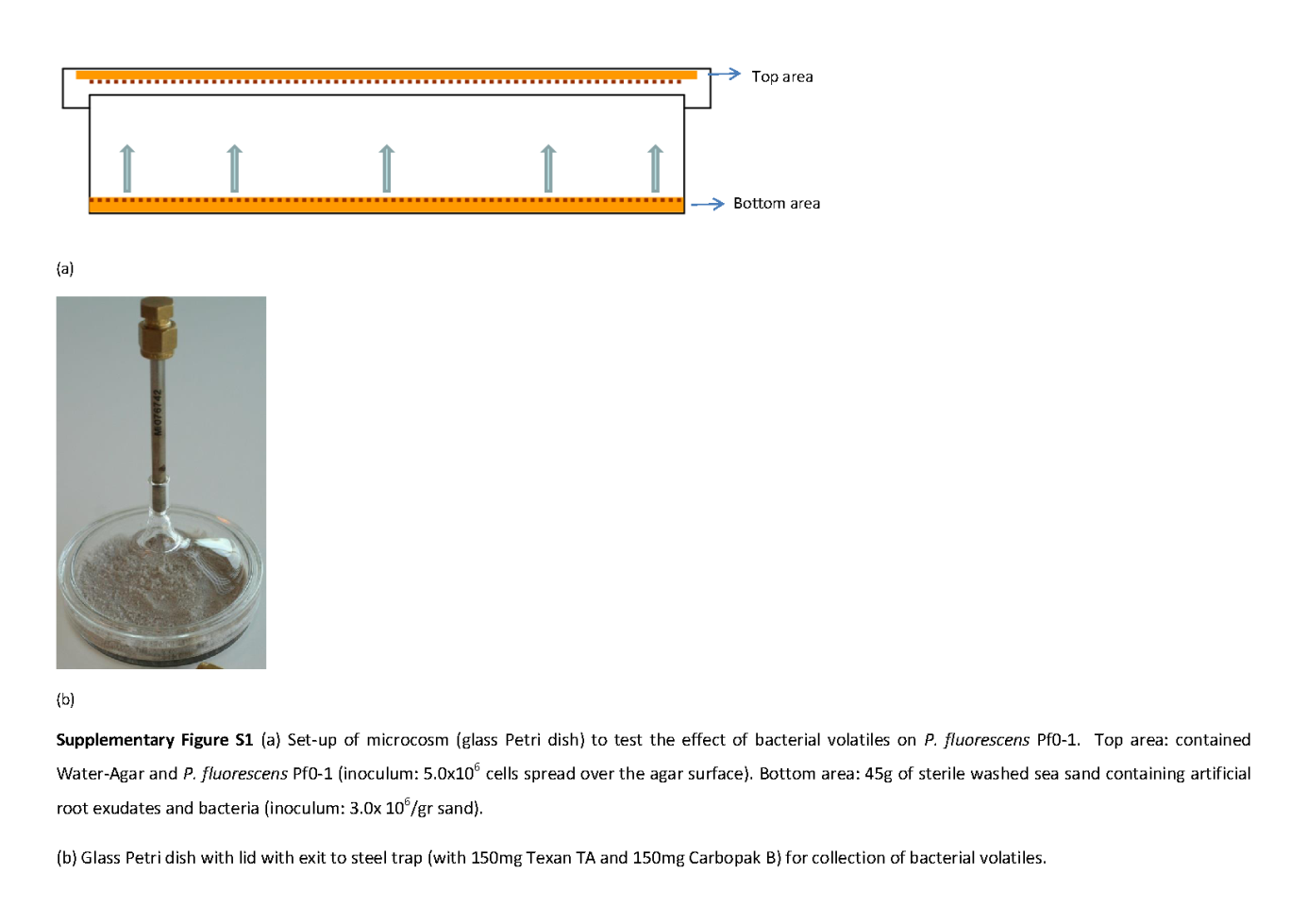





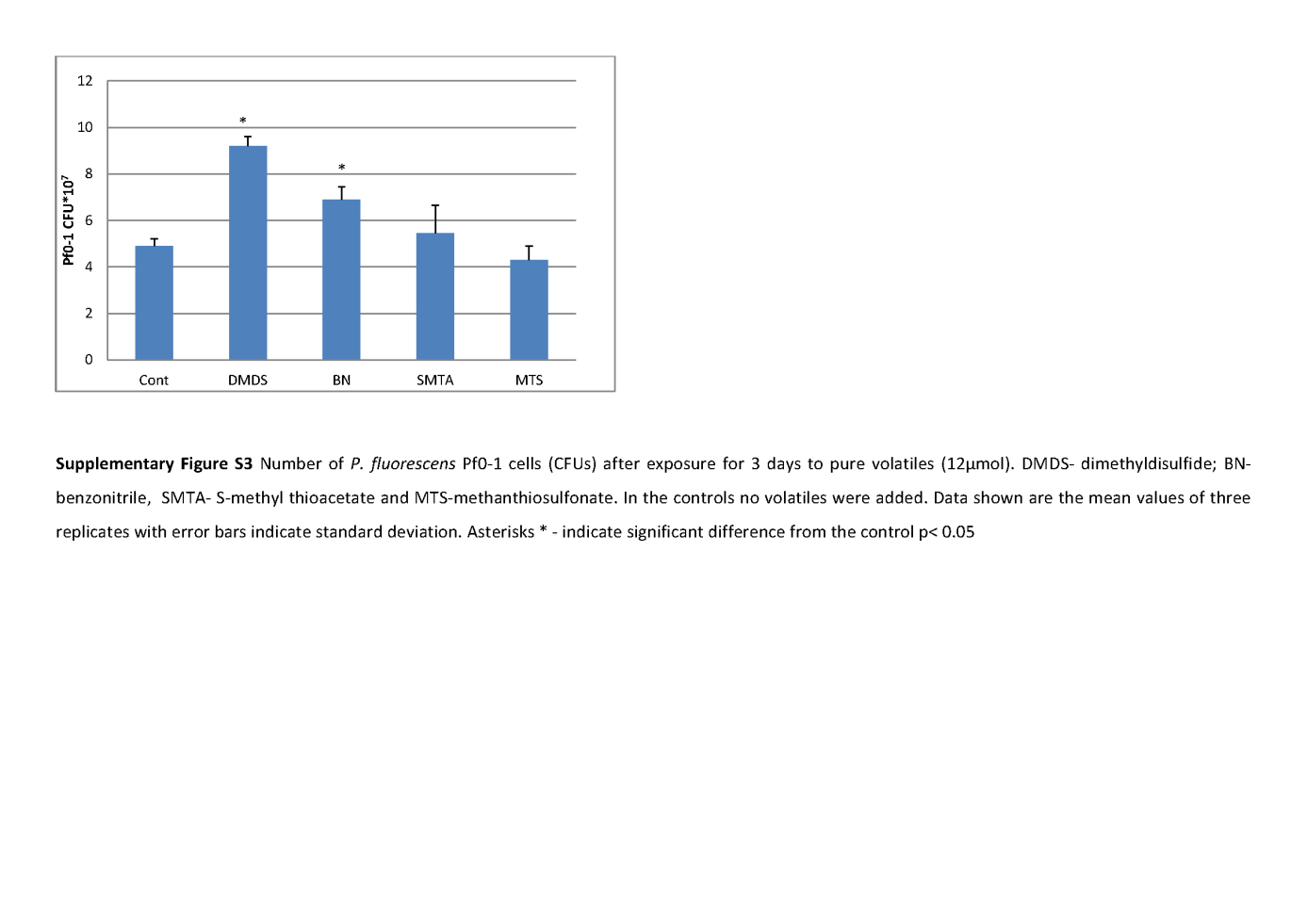


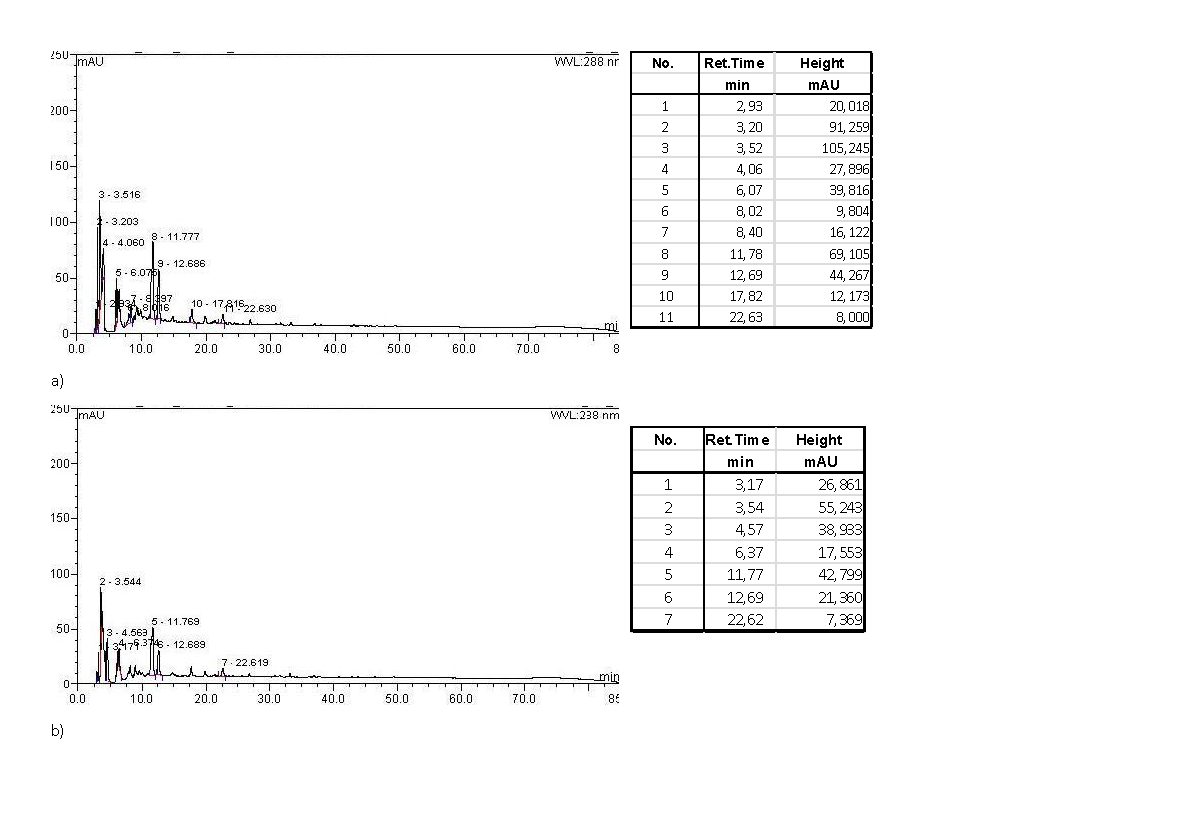

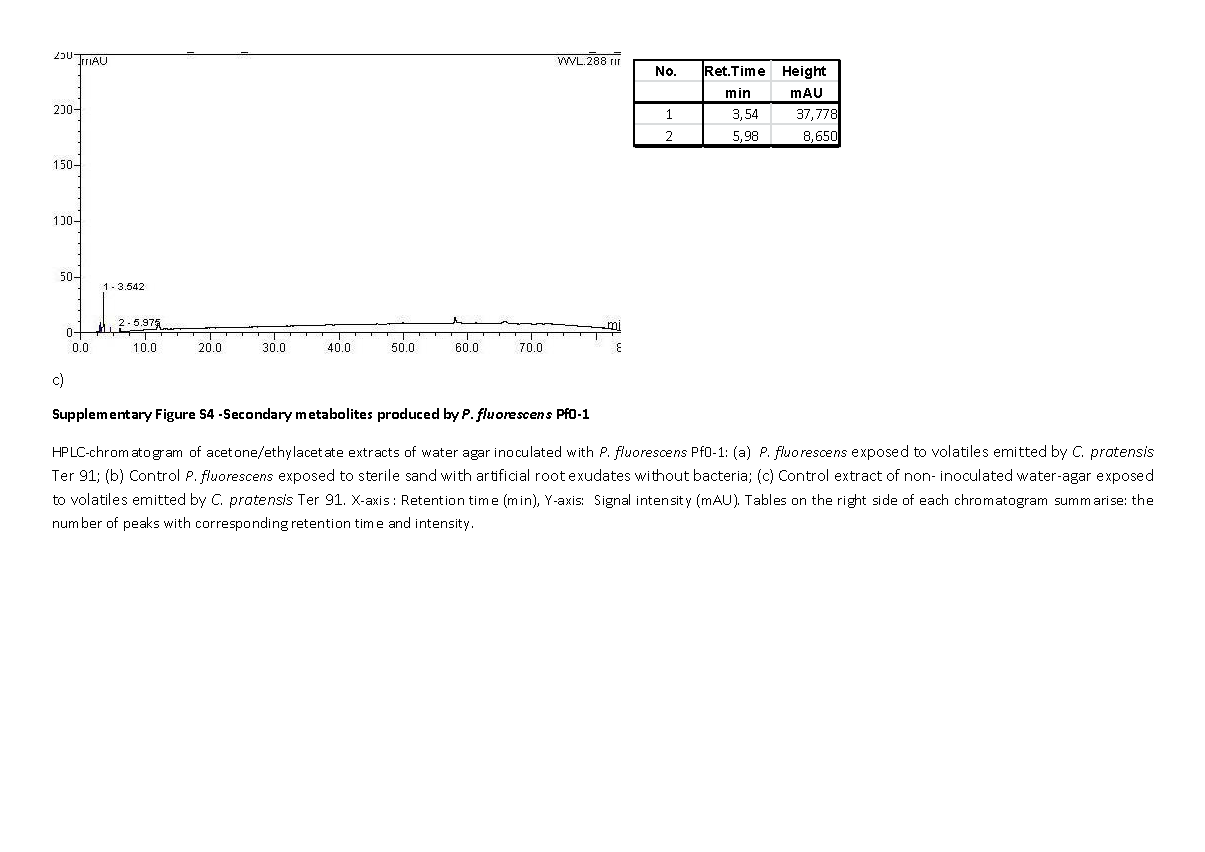

Supplement: Supplementary file 2 [file DataSheet2.DOCX]
